# Density Dependence Promotes Species Coexistence and Provides a Unifying Explanation for Distinct Productivity–Diversity Relationships

**DOI:** 10.1111/ele.70292

**Published:** 2025-12-21

**Authors:** Liang Xu, Christopher A. Klausmeier, Emily Zakem

**Affiliations:** ^1^ Department of Global Ecology, Carnegie Institution for Science Stanford California USA; ^2^ W. K. Kellogg Biological Station, Department of Plant Biology & Integrative Biology, Program in Ecology, Evolution & Behavior Michigan State University East Lansing Michigan USA

**Keywords:** coexistence, density‐dependent mortality, negative density dependence, productivity–diversity curve, resource competition theory, resource consumer model

## Abstract

Understanding diversity patterns in complex communities, such as microbial consortia, requires a mechanistic framework appropriate for many species. Negative density dependence is often utilised in complex ecosystem models, typically as a density‐dependent mortality term for a population, but its full impact on community structure remains unclear. Here we use mechanistic population models of resource consumption to examine the effects of negative density dependence and develop a tractable framework for understanding diversity patterns in complex systems. To provide mechanistic grounding, we quantify how density‐dependent mortality expands coexistence zones along resource gradients in simple communities using graphical analysis. We then derive an analytical formula relating species abundances and coexistence patterns to trait differences among subsets (guilds) of complex communities, in which many species share a resource or predator. Finally, we explain how distinct relationships between productivity and diversity emerge from the resulting mechanistic framework, providing insights into previously unreconciled observed patterns.

## Graphical abstract

We develop a mechanistic framework to understand how negative density dependence shapes diversity in complex communities. By modelling resource consumption and species interactions, we show that density‐dependent mortality expands coexistence zones and explains variable productivity–diversity relationships. A derived formula predicts species abundances within competitive guilds, offering new insight into community structure.

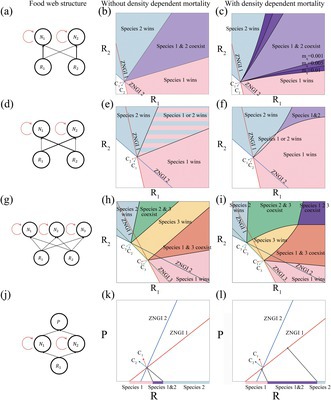

## 1 Introduction

Since Hutchinson articulated the ‘paradox of the plankton’ (Hutchinson 1961), numerous hypotheses have been proposed to explain the coexistence of many plankton species competing for a limited number of resources. Resource competition theory (RCT), a foundational framework in ecology, has deepened our understanding of plankton diversity (Tilman 1982; Litchman and Klausmeier 2001, 2008; Klausmeier et al. 2004). However, RCT analysis is typically restricted to simplified food web structures (Holt 1977; Holt et al. 1994; Chase and Leibold 2003), where its graphical techniques are not easily extended to more than a few (< 3) resources and species. Microbial communities, which contain thousands of species (or species‐like equivalents) consuming diverse substrates (Moran et al. 2022), therefore present a significant challenge to the application of this theoretical framework. To understand species coexistence and diversity patterns in such complex communities, we need a general, comprehensive, and mechanistic framework applicable to diverse systems with many resources and species.

Any such framework must consider the impact of negative density dependence, the suppression of a species' net growth rate as its density increases due to processes such as viral lysis (Winter et al. 2010; Thingstad 2022), host‐specific parasitism (McPeek 1998), and soil pathogen interactions (Bagchi et al. 2014; Comita et al. 2014; LaManna, Belote, et al. 2017; Adler et al. 2018; Tuck et al. 2018). Though phenomenological, density‐dependent mortality in mathematical descriptions of population growth captures processes that are too uncertain or complex to model explicitly, and so is often utilised in studies of complex ecosystems (Bagchi et al. 2014; Fricke and Wright 2017; Adler et al. 2018; Bendik and Dries 2018; Xu et al. 2022). Additionally, density‐dependent mortality is a well‐established mechanism for resolving the paradox of the plankton because it facilitates the persistence of inferior competitors (Chesson 2000; Thingstad 2000).

Although density‐dependent mortality is widely invoked to explain biodiversity patterns (Holt and Lawton 1993; Winter et al. 2010; Bagchi et al. 2014; Comita et al. 2014; LaManna, Mangan, et al. 2017), theoretical studies, grounded in mechanistic resource competition models, remain limited relative to empirical studies. Thus, a key question remains: How does density dependence shape species coexistence and relative abundance patterns along resource gradients? Although theory has shown that density dependence can stabilise coexistence and allow more species to persist than there are limiting resources (Leibold 1998; Adler et al. 2007; McPeek 2012, 2019), most analyses rely on low‐dimensional isocline approaches, leaving its role in more diverse systems poorly understood.

A broader question is how biodiversity responds to productivity and how this relationship varies. Different productivity–diversity relationships (PDRs) have been observed, with monotonic increases and unimodal (hump‐shaped) curves common (Mittelbach et al. 2001). For example, Fraser et al. (2015), analysing 30 grassland sites on six continents, found strong support for a unimodal PDR, with richness peaking at intermediate productivity at regional and global scales. However, Adler et al. (2011) sampled 48 herbaceous plant communities across five continents and found no consistent relationship: while some individual sites showed linear or hump‐shaped trends, the overall pattern lacked coherence.

These observed PDRs are not easily explained by classic resource competition theory alone, since the number of distinct resources sets a limit on the number of stably coexisting species, and it is unlikely that resource diversity increases along productivity gradients at the same pace as species diversity. Additional mechanisms are therefore required. Proposed mechanistic explanations include spatial or temporal heterogeneity (e.g., due to dispersal or time‐varying resource supply) (Abrams 1988; Leibold 1996), sampling limitations (Pastor et al. 1996), and spatial scale dependency (Steiner and Leibold 2004), but any one of these mechanisms cannot explain why multiple distinct patterns may arise. Density‐dependent mortality may constitute another potential mechanistic explanation, but this has not yet been rigorously evaluated. Exploring this potential requires a theoretical framework that can mechanistically link PDRs to trophic interactions and ecosystem structure that is applicable to a large number of species. Ideally, such a framework would also explain how different PDRs may emerge from a general description of ecosystem dynamics.

Here, we develop a unified framework that links density dependence to both species coexistence and PDRs. We begin by extending classic resource competition theory to reveal how density dependence modifies coexistence criteria, reshaping resource consumption vectors and supply points. We use well‐established, simple food web modules (Holt 1997) to provide this mechanistic grounding, illustrating how density dependence enables more species to coexist on limited resources (Section 2). Building on this foundation, we then derive general formulas for species abundances within analytically tractable subsets of a complex community: A bottom‐up (one shared resource) or a top‐down (one shared predator) guild (Section 3). These formulas are applicable to systems with many species, such as diverse microbial consortia with one dominant predator. Finally, we demonstrate how this framework, combined with trait trade‐offs, captures multiple PDRs in consistency with observations, which unifies coexistence theory and PDRs within one mechanistic description (Section 4).

## 2 Graphical Analysis of Simple Systems

We begin by exploring how density‐dependent mortality affects species coexistence in simple systems using graphical analysis, building on previous work (Leibold 1996; Chase and Leibold 2003). Specifically, we quantify how density‐dependent mortality expands the coexistence domain in three well‐known food web modules: (Abrams 1988) two species competing for two substitutable resources, (Adler et al. 2007) three generalists competing for two resources, and (Adler et al. 2011) a diamond food web with two prey species and one shared predator.

### 2.1 Two Species and Two Resources

We first consider a food web of two species competing for two nutritionally substitutable resources. Assuming linear functional responses, species i with density $N_{i}$ grows at rate $\mu_{\mathrm{ij}}$ on resources $R_{j}$, supplied with concentration $s_{j}$ and dilution rate a:

(1a) $$\frac{1}{N_{1}}\frac{dN_{1}}{\mathrm{dt}}=\mu_{11}R_{1}+\mu_{12}R_{2}-m-m_{q}N_{1}$$

(1b) $$\begin{array}{c} \frac{1}{N_{2}}\frac{dN_{2}}{\mathrm{dt}}=\mu_{21}R_{1}+\mu_{22}R_{2}-m-m_{q}N_{2}\; \end{array}$$

(1c) $$\begin{array}{c} \frac{dR_{1}}{\mathrm{dt}}=a\left(s_{1}-R_{1}\right)-V_{11}N_{1}R_{1}-V_{21}N_{2}R_{1}\; \end{array}$$

(1d) $$\begin{array}{c} \frac{dR_{2}}{\mathrm{dt}}=a\left(s_{2}-R_{2}\right)-V_{12}N_{1}R_{2}-V_{22}N_{2}R_{2}. \end{array}$$

Here $V_{\mathrm{ij}}$ are consumption rates with yields $Y_{\mathrm{ij}}=\mu_{\mathrm{ij}}/V_{\mathrm{ij}}$. Density‐dependent mortality for species i with density *N*_*i*_ can be modelled as $m_{q}{N_{i}}^{x-1},\text{where}\;x>1$. For simplicity, we use quadratic mortality (x=2) in the main text. We generally assume equal linear mortality rates (m) and quadratic mortality constants ($m_{q}$). Species‐specific cases can be rescaled to the general framework, providing quantitative but not qualitative variability to results (Supporting Information).

**FIGURE 1:**
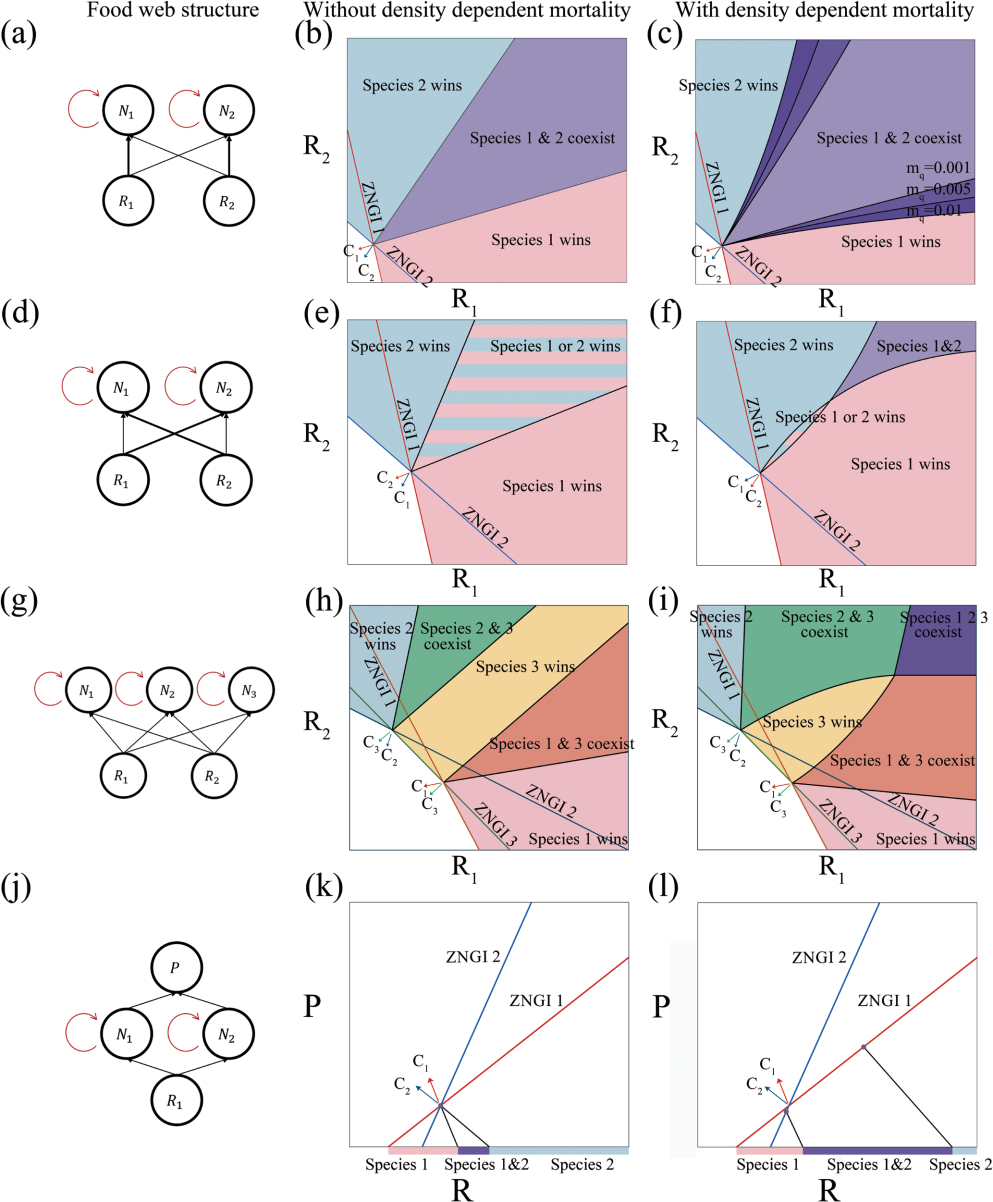
Illustration of the range of resource supply rates for the species coexistence with and without the density‐dependent effect. The impact vectors of species on resources are denoted by $c_{1},c_{2},c_{3}$. (a, d, g, j). The food web structures. (b) The two generalists can coexist when $m_{q}=0;$ (d). Increasing $m_{q}$ expands the range of resource supply rates for species coexistence. The model parameters are $\mu_{11}=1.0,\mu_{12}=0.2,\mu_{21}=0.5,\mu_{22}=0.5,V_{11}=3.3,V_{12}=0.67,V_{21}=1.67,V_{22}=1.67,m=0.1,a=1;$ (e). The two generalists cannot coexist when $m_{q}=0$ because their impact vectors indicate a priority effect; (f). The effect of density dependence facilitates coexistence at higher supply rates. For the supply rates vector falls in the striped zone, the priority effect emerges. The model parameters are $\mu_{11}=1.0,\mu_{12}=0.2,\mu_{21}=0.5,\mu_{22}=0.5,V_{11}=1.1,V_{12}=1.8,V_{21}=2.2,V_{22}=0.6,m=0.1,a=1.$ (h). When $m_{q}=0$, at most two species can coexist in the supply rate space. The outcome of species interaction depends on where the vector of resource productivities falls.; (i). When $m_{q}=0.01$, the two pairwise coexistence zones overlap, indicating an area where three species can coexist. The model parameters are $a=1,\mu_{11}=0.165,\mu_{12}=0.069,\mu_{21}=0.072,\mu_{22}=0.171,\mu_{31}=0.15,\mu_{32}=0.15,V_{11}=0.55,V_{12}=0.23,V_{21}=0.24,V_{22}=0.57,V_{31}=0.5,V_{32}=0.5,m=0.1,a=1.$ (k–l). In the diamond food web, two species can coexist at a larger range of supply rates with increase of $m_{q};$ (k).$m_{q}=0$; (l). $m_{q}=0.01$; The boundaries are given by Equations (4) and (5). The model parameters are $\mu_{1}=2.8,\mu_{2}=1.2,V_{1}=4.2,V_{2}=1.8,g_{1}=8.7,g_{2}=1.725,f_{1}=5.8,f_{2}=1.15,m=0.1,m_{p}=0.1,a=1.$

Without density‐dependent mortality ($m_{q}=0$), coexistence requires each species to be competitively superior on one limiting resource, represented by intersecting ZNGIs (zero net growth isoclines) (Ryabov and Blasius 2011; McPeek 2014; Kleinhesselink and Adler 2015; Letten et al. 2017) (Figure 1b). Density‐dependent mortality does not alter invasion criteria—resource levels must still lie above a species' invasion ZNGI where $N_{i}=0$—but shifts equilibrium resource levels (${\hat{N}}_{i}>0$) to depend on species densities and thus on supply rates. Equilibrium ZNGIs are:

(2a) $$\mu_{11}{\hat{R}}_{1}+\mu_{12}{\hat{R}}_{2}=m+m_{q}{\hat{N}}_{1}$$

(2b) $$\mu_{21}{\hat{R}}_{1}+\mu_{22}{\hat{R}}_{2}=m+m_{q}{\hat{N}}_{2}$$

where equilibrium conditions are denoted by $\hat{∎}$'s. Unlike invasion ZNGIs, these equilibrium ZNGIs with ${\hat{N}}_{1}>0$ vary with biomass and elevate equilibrium resource levels (Figure S2). As supply increases, mortality rises with density, raising resources. Strong competitors benefit from increased growth but also suffer higher mortality, while weaker ones, at lower densities, experience less mortality. This asymmetry broadens the coexistence domain beyond what is possible under pure resource competition (Leibold 1998; McPeek 2019).

We next quantitatively analyse the impact of density‐dependent mortality on the boundaries between competitive exclusion and coexistence regions using mutual invasibility analysis (Chesson 2000) (Figure 1c) (Supporting Information). Density‐dependent mortality significantly widens coexistence ranges and produces nonlinear boundaries between competitive exclusion and coexistence regions (Figure 1c). The lower boundary of the coexistence zone curves downward compared to the curve of $m_{q}=0$. This indicates that species 2 can invade at a lower supply rate of resource 2 when species 1 dominates, since species 1 cannot deplete resources to its invasion ZNGI ($\mu_{11}R_{1}+\mu_{12}R_{2}=m$) but only to the higher equilibrium ZNGI $\mu_{11}{\hat{R}}_{1}+\mu_{12}{\hat{R}}_{2}=m+m_{q}{\hat{N}}_{1}$. The same principle applies to the upper boundary of the coexistence zone when species 1 can invade a community with the resident species 2.

In general, a larger $m_{q}$ results in a larger coexistence zone, indicating that stronger density‐dependent mortality promotes species coexistence across an expanded range of resource supply rates (Figure 1c). Box 1 summarises the four possible competitive outcomes.

BOX 1 1 In a food web with two species competing for two nutritionally substitutable resources, density‐dependent mortality affects the four regions of the resource plane as follows (Figure 1a–c):

- Region of no species: This zone is bounded by the invasion ZNGIs of the two species and two resource axes. In this zone, the supply of both resources is severely limited and cannot support the persistence of either species. This zone is not changed when incorporating density‐dependent mortality because the minimum requirement of resources is not impacted: the invasion ZNGIs remain constant as Equation (1), do not depend on $m_{q}$ when $N_{i}=0$.
- Regions of competitive exclusion: In these regions, resource 1 (or 2) is sufficiently supplied, which allows species 1 (or 2) to maintain its presence. In contrast, the limited supply of resource 2 (or 1) hinders the survival of species 2 (or 1), resulting in its exclusion. The presence of density‐dependent mortality narrows this zone and allows species 2 (or 1) to eventually persist with the increase of the supply concentration of resource 2 (or 1).
- Region of coexistence: This region is bounded by two boundaries characterized by adequate resource supply levels for both resources. Consequently, it creates an environment where both species can coexist. Density‐dependent mortality significantly expands this zone (Figure 1c).

In a food web with two species *N*_*i*_ competing for one resource *R* and consumed by a common predator *P*, density‐dependent mortality affects the four regions of the resource plane as follows (Figure 1j–l):

- Region of no species: When $s<R_{1}^{*}=\frac{m}{\mu_{1}}$, resource supply is insufficient to meet the minimum requirement for either species, even for the best resource exploiter. No species can persist in this range of resource supply rate. Incorporating density‐dependent mortality does not change this threshold.
- Region of species 1 winning: When $R_{1}^{*}<s<s_{21}$, species 1 can persist, but not species 2. Specifically, in the range of $R_{1}^{*}<s<R_{2}^{*}=\frac{m}{\mu_{2}}$, species 2 cannot persist because the resource supply concentration is below its minimum requirement. When $R_{2}^{*}\leq s<s_{21}$, species 2 is excluded by competition with species 1. Density‐dependent mortality decreases $s_{21}$ (Equation 4), which allows species 2 to invade at a lower resource supply compared to the case of no density‐dependent mortality.
- Region of coexistence: When $s_{21}<s<s_{12}$, both species can coexist. The effect of density‐dependent mortality expands the coexistence region through increasing $s_{12}$ and decreasing $s_{21}$ with increasing $m_{q}$ (Figure 1k,l).
- Region of species 2 winning: In the last region where $s>s_{12}$, only species 2 can survive. Increasing resource supply raises the total density of consumer species, resulting in an increase in the density of the shared predator. This apparent competition leads to the exclusion of species 1.

### 2.2 Density‐dependent mortality reduces the likelihood of priority effects

Notably, density‐dependent mortality allows coexistence where two generalists would otherwise exhibit priority effects (Chesson 2000; Fukami 2015; Grainger et al. 2019). In Figure 1d–f, we selected consumption rates for the two species where each species consumes more of its less preferred resource ($\frac{V_{12}}{V_{11}}>\frac{V_{22}}{V_{21}}$), normally yielding first‐come‐first‐survive without density‐dependent mortality. The striped area on the resource plane in Figure 1e represents where priority effects occur: the species that initially establishes itself in the community excludes the other. Without density‐dependent mortality, this region expands linearly with resource supply, with no intersection of the boundaries (Figure 1e). In contrast, with density‐dependent mortality, the invasion boundaries intersect at sufficiently high resource supply levels (Figure 1f). Thus, low supply maintains the priority effect, but high supply enables coexistence. This finding suggests that both supply magnitude and ratio govern the ecological outcomes. The regions with uniform colour in Figure 1e,f continue to represent dominance by a single species, suggesting that competitive exclusion remains possible under uneven resource supply ratios.

### 2.3 Three Species and Two Resources

We next examine how density‐dependent mortality can expand not only the domain of coexistence but also increase the total number of coexisting species. We consider a food web with three species and two substitutable resources, with two relatively specialised consumers (species 1 and 2) with advantages on different resources, and one generalist (species 3) having intermediate advantages on both resources. Without density‐dependent mortality, the system exhibits two distinct regions of pairwise coexistence. As in the above example, density‐dependent mortality expands the coexistence regions by bending the invasion boundaries (Figure 1g–i). In this case, the two coexistence zones may overlap at high resource supply rates, leading to coexistence of all three species (Figure 1i).

### 2.4 The Diamond Food Web: Two Species, One Predator, and One Resource

We finally demonstrate how density‐dependent mortality promotes species coexistence in a different classic system: The ‘diamond’ food web with two species *N*_*i*_ competing for one resource *R* and consumed by a common predator P (apparent competition (Holt 1977))as:

(3a) $$\begin{array}{c} \frac{1}{P}\frac{\mathrm{dP}}{\mathrm{dt}}=f_{1}N_{1}+f_{2}N_{2}-m_{p} \end{array}$$

(3b) $$\begin{array}{c} \frac{1}{N_{1}}\frac{dN_{1}}{\mathrm{dt}}=\mu_{1}R-m-g_{1}P-m_{q}N_{1} \end{array}$$

(3c) $$\begin{array}{c} \frac{1}{N_{2}}\frac{dN_{2}}{\mathrm{dt}}=\mu_{2}R-m-g_{2}P-m_{q}N_{2}\; \end{array}$$

(3d) $$\begin{array}{c} \frac{\mathrm{dR}}{\mathrm{dt}}=a\left(s-R\right)-V_{1}N_{1}R-V_{2}N_{2}R, \end{array}$$

where $f_{i}$ is predator growth at predation rate $g_{i}$ on species i, and $m_{p}$ denotes the mortality rate of the predator. Without density‐dependent mortality, coexistence requires the two species' ZNGIs to intersect on the predator‐resource plane (Figure 1k). The stability of this equilibrium depends on the condition that the species with superior resource–competitive capabilities is more poorly defended against the predator, while the worse resource competitor is better‐defended against the predator (Leibold 1996). Here, we assume that species 1 is the better resource competitor and species 2 is better defended against the predator, that is, $\mu_{1}>\mu_{2},g_{1}>g_{2}$.

With density‐dependent mortality, the two critical resource supply concentrations are given by:

(4) $$\begin{array}{c} s_{21}=\frac{\left(g_{1}-g_{2}\right)m-m_{q}\frac{m_{p}g_{2}}{f_{1}}}{g_{1}\mu_{2}-g_{2}\mu_{1}}\left(1+\frac{V_{1}m_{p}}{af_{1}}\right), \end{array}$$

and

(5) $$\begin{array}{c} s_{12}=\frac{\left(g_{1}-g_{2}\right)m+m_{q}\frac{m_{p}g_{1}}{f_{2}}}{g_{1}\mu_{2}-g_{2}\mu_{1}}\left(1+\frac{V_{2}m_{p}}{{\mathrm{af}}_{2}}\right) \end{array}$$

where $s_{\mathrm{ij}}$ denotes the threshold of the supply rate for species i invading species j (Supporting Information). Coexistence is possible when the resource supply s falls between these two threshold values. Increasing $m_{q}$ increases the interval between $s_{21}$ and $s_{12}$, promoting coexistence. We note that the invasion point of species 2 is still bounded by a minimum supply, which is determined by the intercept of its ZNGI and the supply axis. Unlike the previous cases (Figure 1a–i), further increasing resource supply does not necessarily result in coexistence, as it ultimately goes beyond the coexistence interval, favouring the superior apparent competitor, species 2 (Figure 1L). Thus, although density dependence continues to promote species coexistence, its effect is weakened by apparent competition as resource supply increases. The community composition then shifts towards species with higher predator resistance.

Note that the case of no density‐dependent mortality ($m_{q}=0$) is a special case of these general equations (Supporting Information). The four possible competitive outcomes are summarised in Box 1 (Figure 1i–l).

## 3 Control Formulas for Complex Systems

We have quantified how density‐dependent effects promote species coexistence, allowing more species to coexist than the number of limiting factors in simple systems. However, graphical analysis becomes impractical for quantitatively extending this approach to communities with more than a few species. We need a more general quantitative approach to mechanistically understand how diversity changes with resource productivity across different food‐web structures in more complex communities.

**FIGURE 2:**
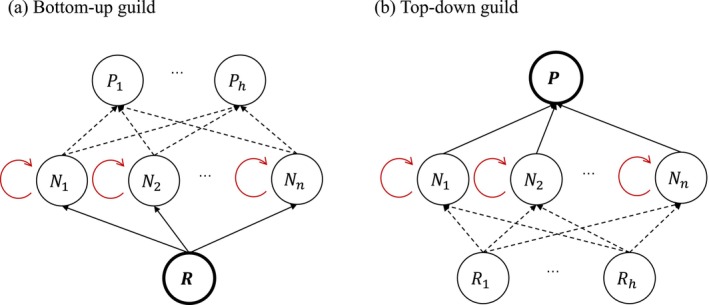
Two types of guilds illustrating species interactions within and between trophic levels. The negative density dependence is denoted by the red arrows. (a) The bottom‐up guild assumes that consumer species are mainly limited by food availability of one common resource; (b) The top‐down guild assumes that consumer species are restricted by the common predator. Predators are denoted by P; consumer species are denoted by N; resources are denoted by R.

To address this, we consider analytically tractable subsets of communities with many coexisting species. Specifically, we consider two types of subsets, which we refer to as guilds: (Abrams 1988) a bottom‐up guild, in which multiple species compete for a single shared resource, while the apparent competition is negligible (Figure 2a); and (Adler et al. 2007) a top‐down guild, in which multiple species are preyed upon and mainly constrained by a single shared predator, without details of their resource use (Figure 2b). This approach aligns with a common and ecologically realistic strategy of analysing food‐web modules—simplified subnetworks where species interactions are governed by basic trophic relationships, such as resource consumption and predation (Holt 1997)—while still allowing considerable complexity in the rest of the food web. For example, tropical forests, which harbour high plant diversity competing for similar resources (Gentry 1988), and marine phytoplankton, which compete for a limited set of inorganic nutrients (Hutchinson 1961), both exemplify bottom‐up guilds. Top‐down control is evident in freshwater lakes, where invertebrate and vertebrate predators structure distinct prey assemblages (Werner and McPeek 1994). Similarly, microorganisms of comparable size often share predators due to size‐based predation (Clauset and Erwin 2008; Ward et al. 2012; Follett et al. 2022). Considering these guilds, each with a single shared limiting factor, permits analytical treatment that would be intractable in fully specified complex networks.

We next develop analytical expressions to characterise coexistence patterns of these guilds, which allows us to later explore mechanistic relationships between productivity and diversity. We present a general control formula for a species—where its abundance is mainly controlled (constrained) by either resource availability or predation pressure—that results from our derivations of the two guild‐specific control formulas. This general form provides ecological insight into coexistence dynamics, including the impact of density‐dependent mortality (Section 3.1). We then present the specifics of the bottom‐up formula (Section 3.2), noting that the corresponding specifics of the top‐down formula follow a similar derivation and structure (Box 2). We use the bottom‐up formula to link coexistence and productivity–diversity patterns within one framework (Section 3.3).

BOX 2 **The control formula derivation and detail.**

1 Derivation overview: There are two ways to derive the density of species at equilibrium. The most direct approach is to solve Equation (7a,b) for equilibrium. Specifically, at equilibrium, the density of the consumer can be obtained by setting Equation (7a) to 0:

(12) $$\begin{array}{c} N_{i}=\frac{1}{m_{q}}\left(\mu_{i}R-m-\sum_{h=1}^{l}g_{\mathrm{hi}}P_{h}\right)=\frac{G_{i}}{m_{q}} \end{array}$$

This equation shows that consumer density increases with gains from resources but decreases due to linear mortality and predation. Moreover, density is inversely related to the nonlinear mortality rate. While this formulation is broadly applicable in general food webs, it does not reveal how interactions among species within trophic levels contribute to changes in density.

To address this, we instead apply a contrast‐based decomposition. In this approach, we derive formulas specifically for the bottom‐up and top‐down guilds (see Supporting Information Section 1) and then present a general control expression as the shared functional form. The resulting equation differs from Equation (12)—particularly in the weighting terms. The advantage of this derivation – our control formula – provides a richer ecological interpretation, as discussed in the main text and summarized in Equations (8) and (15).

The control formula for top‐down trophic guilds: Both the bottom‐up and top‐down guild control formula have the same functional form, the general control formula. In the main text, we provide the specifics for the bottom‐up guild, and here we provide the analogues specifics for the tow‐down guild. In a top‐down trophic guild, all species are preyed upon and largely controlled by a common predator, with unspecified resources that do not constrain total density of consumer species, given by (Figure 2b):

(13) $$\begin{array}{c} \frac{1}{P}\frac{\mathrm{dP}}{\mathrm{dt}}=\sum_{i=1}^{n}f_{i}N_{i}-m_{p} \end{array}$$

(14) $$\begin{array}{c} \frac{1}{N_{i}}\frac{dN_{i}}{\mathrm{dt}}=\sum_{h=1}^{k}\mu_{\mathrm{ih}}R_{h}-g_{i}P-m-m_{q}N_{i} \end{array}$$

In this case, the explicit form of the control formula Equation (7) is given by

(15) $$\begin{array}{c} {\hat{N}}_{i}=\frac{m_{p}}{\sum_{j=1}^{n}f_{j}}+\frac{1}{m_{q}}\left(G_{i}-\frac{\sum_{j=1}^{n}f_{j}G_{j}}{\sum_{j=1}^{n}f_{j}}\right) \end{array}$$

where the average biomass density reflects the control from the predator as $\bar{N}=\frac{m_{p}}{\sum_{j=1}^{n}f_{j}}=\frac{\sum_{j=1}^{n}f_{j}{\hat{N}}_{j}}{\sum_{j=1}^{n}f_{j}}$, which is the subsistence concentration of the predator, or the mean density ${\hat{N}}_{j}$weighted by the predation rate $f_{j}$. The average community invasion growth rate is weighted by the predator's assimilation (consumption) rate on each species as $\bar{G}=\frac{\sum_{j=1}^{n}f_{j}G_{j}}{\sum_{j=1}^{n}f_{j}}$, where $G_{i}=\sum_{h=1}^{k}\mu_{\mathrm{ih}}R_{h}-g_{i}P-m$. Note that we assume a static (non‐oscillatory) equilibrium state, though oscillatory equilibria can be treated by averaging species densities over cycles.

### 3.1 The General Control Formula

We derive control formulas for each of the guilds, which results in one general control formula for species abundances (see Box 2 for derivation overview and SM Section 1). The resulting general formula describes the steady‐state density ${\hat{N}}_{i}$ of species i as:

(6) $$\begin{array}{c} {\hat{N}}_{i}=\bar{N}+\frac{\left(G_{i}-\bar{G}\right)}{m_{q}}\\ =\left[\text{average equilibrium density}\right]+\frac{\left[\;\text{growth advantage}/\text{disadvantage of species}\;i\right]}{\left[\text{strength of density}-\text{dependent mortality}\right]}\; \end{array}$$

where $G_{i}$ is the net per‐capita growth rate of species i excluding density‐dependent losses, which we refer to the invasion growth rate. $\bar{G}$ is the community‐average growth rate, and $\bar{N}$ denotes the mean equilibrium density, which is determined by the limiting factor (either the resource or the predator).

This expression captures how species abundances depend jointly on the difference between their growth advantages from the rest of the community and the strength of density regulation, in addition to the overall limitation on biomass imposed by the resource or the predator. This also links differences in traits (which shape *G*) between species and the broader community to coexistence and thus diversity patterns. When density dependence is weak ($m_{q}\to 0$), coexistence requires that all coexisting species have equal invasion growth rates ($G_{i}=\bar{G}$) at equilibrium; as density dependence strengthens, species with slightly lower growth rates can persist, thereby promoting diversity.

### 3.2 The Specific Control Formula for a Bottom‐Up Guild

In a bottom‐up guild, multiple consumer species compete for and are predominantly limited by a single shared resource. Predation is not the main constraint on total consumer density, that is, apparent competition is negligible (Figure 2a). The system is described as:

(7a) $$\begin{array}{c} \frac{1}{N_{i}}\frac{dN_{i}}{\mathrm{dt}}=\mu_{i}R-m-\sum_{h=1}^{l}g_{\mathrm{hi}}P_{h}-m_{q}N_{i}\; \end{array}$$

(7b) $$\begin{array}{c} \frac{\mathrm{dR}}{\mathrm{dt}}=a\left(s-R\right)-\sum_{i=1}^{n}V_{i}N_{i}R \end{array}$$

with parameters as defined above (Equation 1). The specific form of the control formula (Equation 6) for the bottom‐up guild is.

(8) $${\hat{N}}_{i}=\frac{a\left(s-\hat{R}\right)}{\sum_{j=1}^{n}V_{j}\hat{R}}+\frac{1}{m_{q}}\left(G_{i}-\frac{\sum_{j=1}^{n}V_{j}G_{j}}{\sum_{j=1}^{n}V_{j}}\right)$$

where the average density is $\bar{N}=\frac{a\left(s-\hat{R}\right)}{\sum_{j=1}^{n}V_{j}\hat{R}}=\frac{\sum_{j=1}^{n}V_{j}{\hat{N}}_{j}}{\sum_{j=1}^{n}V_{j}}$, representing the total resource flux ($a\left(s-\hat{R}\right)$) divided by the total uptake rate of all sustained species ($\sum_{j=1}^{n}V_{j}\hat{R}$). The average community growth rate is weighted by each species' resource uptake rate $\bar{G}=\frac{\sum_{j}^{n}V_{j}G_{j}}{\sum_{j}^{n}V_{j}}$, where $G_{j}=\mu_{j}R-m-\sum_{h=1}^{l}g_{\mathrm{hj}}P_{h}$.

### 3.3 Linking Species Persistence and Productivity

Species persistence follows directly from the invasion condition, G>0, evaluated when the focal species is rare (${\hat{N}}_{i}\approx 0$) and the resident community is at equilibrium. The functional dependence of G on the external resource supply s determines how species diversity responds along a productivity gradient. Generally, the equilibrium resource concentration $\hat{R}$ increases monotonically with the external resource supply s (Figure S3). However, increased $\hat{R}$ also strengthens apparent competition by increasing the density of common predators in a top‐down guild, consequently leading to species exclusion. The key question, then, is how increasing the external resource supply s influences the invasion growth rate G. In the following section, we show that the impact of predation, via apparent competition, results in a distinct relationship between G and s compared to a system in which it is negligible, giving rise to contrasting productivity–diversity patterns.

## 4 Mechanistic Explanations for Distinct Productivity–Diversity Relationships

Our mechanistic framework links resource supply rate, which governs the productivity of the ecosystem, to species richness and diversity. Here, we explore how this provides a mechanistic basis for the two most commonly observed PDRs: increasing and unimodal. We identify the factors that determine whether one or the other should emerge and use our results to interpret observations in new ways.

### 4.1 Increasing Productivity–Diversity Relationship

We continue to use the control formula, specific for a bottom‐up guild, to show how diversity increases with productivity via the resource supply rate. Critically, this makes the assumption that shared predation has negligible impact on species coexistence patterns. This may, for example, describe phytoplankton communities competing for scarce nutrients with little grazing pressure in oligotrophic systems (Reynolds 1984).

We illustrate the ecological dynamics underlying the increasing PDR clearly by using a specific example of this guild structure with simple tradeoffs between species traits. In a purely bottom‐up guild with negligible predation, the invasion growth rate is $G_{i}=\mu_{i}R-m$, which is a monotonically increasing function of the equilibrium resource concentration $\hat{R}$. With density‐dependent mortality ($m_{q}>0$), the equilibrium resource concentration increases with the supply rate (Figures S1 and S3):

(9) $$\begin{array}{c} \frac{d\hat{R}}{\mathrm{ds}}>0 \end{array}$$

which in turn gives

(10) $$\begin{array}{c} \frac{dG_{i}}{\mathrm{ds}}=\mu_{i}\frac{d\hat{R}}{\mathrm{ds}}>0 \end{array}$$

**FIGURE 3:**
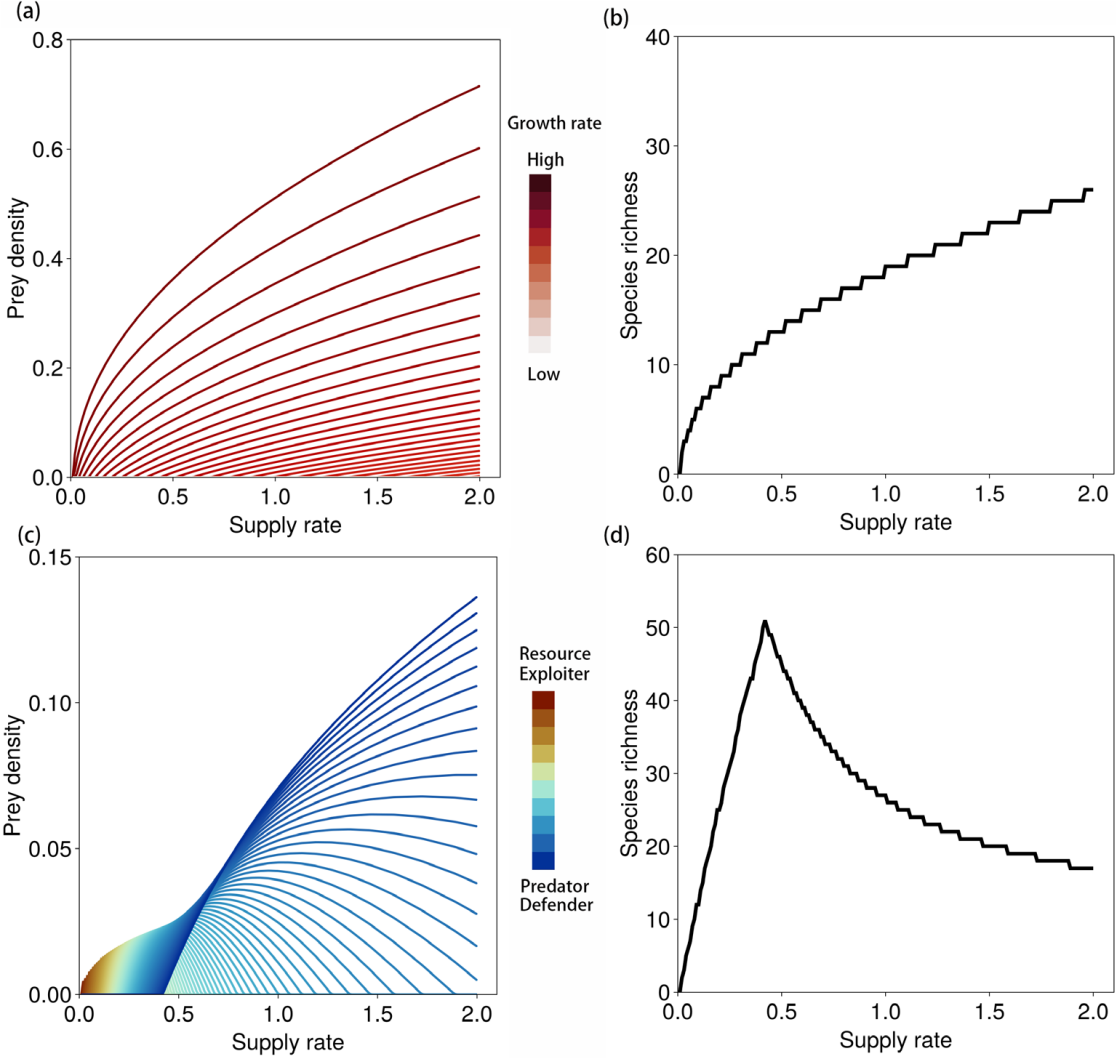
Negative density dependence generates contrasting productivity–diversity relationships. (a) Simulation of a bottom‐up guild with 100 species ranked by growth rates (colour gradient). (b) In the presence of density‐dependent mortality, species richness increases with resource supply. (c) Simulation of a diamond food web with 100 species, sharing a common predator and resource. Species are ranked by growth and predation rates. (d) Under density‐dependent mortality, the resulting productivity–diversity relationship becomes unimodal. As in Section 2, species are ordered by decreasing intrinsic growth rates $\left(\mu_{i}>\mu_{i+1}>\cdots >\mu_{k}\right)$ for simulations in (a, b) and with additional condition $\frac{\mu_{i}}{g_{i}}<\cdots <\frac{\mu_{k}}{g_{k}}$ for simulations in (c, d).

Thus, $G_{i}$ increases with the supply rate, indicating species i can always persist once it has successfully invaded. This mechanism produces an increasing PDR (Figure 3b).

**FIGURE 4:**
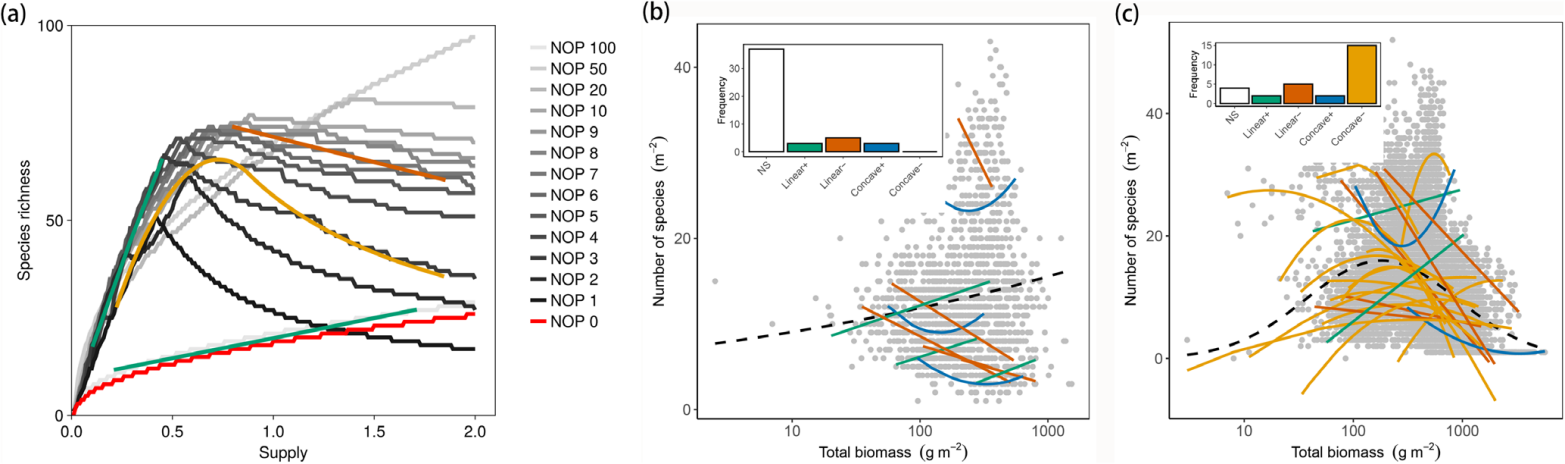
Density‐dependent mortality generates productivity–diversity relationships (PDRs). (a) Simulated PDRs in a food web with 100 prey species and varying number of predators (NOP = 0,…, 100). Different PDR shapes emerge at different ranges of resource supply rates (indicated by coloured lines). Solid green lines: Increasing PDRs; solid yellow lines: Unimodal PDRs; solid brown lines: Decreasing PDRs; solid blue lines: Concave‐up PDRs. (b, c) Empirical PDRs compiled from Adler et al. (2011) and Fraser et al. (2015) (see Supporting Information). Solid lines show within‐site regressions; dashed lines indicate regressions pooled across sites. Inset panels show the distribution of observed PDR types: Concave−, unimodal relationships; Concave+, antiunimodal; Linear−, negative linear; Linear+, positive linear; NS, nonsignificant.

More generally, this framework indicates that an increasing PDR is expected whenever the invasion growth rate G remains stable or increases with productivity for the majority of species. This outcome is fundamentally shaped by density‐dependent mortality, which acts as the key regulatory mechanism enabling the pattern. Importantly, this outcome is not restricted to a purely bottom‐up guild without predators. Even when predation is present, increasing PDRs can still emerge provided that apparent competition is weak, such as if predation is sufficiently specialised to avoid driving species to extinction through shared predators (Holt 1977). Thus, increasing PDRs arise not only in pure bottom‐up systems but also in predator–prey systems with specialised or low rates of predation (Figure 4a, Supporting Information Section 3.5). We next explore how apparent competition via predation qualitatively changes the shape of PDR.

### 4.2 Unimodal Productivity–Diversity Relationship

We illustrate how a qualitatively different, unimodal PDR emerges from the same general description of ecosystem dynamics, but when the details regarding predation are different. Specifically, we add a shared predator to the above bottom‐up guild, resulting in the diamond food web structure, the simple but ecologically relevant case where prey species share a single resource and a common predator (explored using graphical analysis in Section 2.4) (Levin 1970; Holt et al. 1994; Leibold 1996). Thus, in contrast to the bottom‐up guild, apparent competition from shared predation is significant. As in Section 2.4, we assume a trade‐off between intrinsic growth rate and vulnerability to predation, reflecting a range of strategies from efficient resource exploiters (high μ, high g) to predator‐resistant species (low μ, low g).

In this system, the invasion growth rate $G_{i}=\mu_{i}R-m-g_{i}P$ is no longer monotonic with resource supply rate. The derivative of $G_{i}$ with respect to s takes the form

(11) $$\begin{array}{c} \frac{dG_{i}}{\mathrm{ds}}=\left(\mu_{i}-g_{i}\frac{\sum_{j}f_{j}\mu_{j}}{\sum_{j}f_{j}g_{j}}\right)\cdot \frac{d\hat{R}}{\mathrm{ds}} \end{array}$$

indicating that G exhibits variable responses to changes in the supply rate s and species diversity (Supporting Information).

As above, increasing the supply rate increases the resource concentration, allowing more species to invade. However, the term $\mu_{i}-g_{i}\frac{\sum_{j}f_{j}\mu_{j}}{\sum_{j}f_{j}g_{j}}$ decreases when more species that are more resistant to the predator (with sequentially smaller μ and g than the $i^{\mathrm{th}}$ species) invade (Supporting Information). Consequently, as the resource supply rate continues to increase, more predation‐resistant species are established, reducing $G_{i}$. Eventually, $G_{i}$ becomes negative, leading to the exclusion of fast‐growing but predator‐vulnerable species. This process unfolds sequentially along the supply gradient.

Meanwhile, a shared predator can only persist once total prey biomass exceeds a threshold, $\sum_{i}f_{i}N_{i}>m_{p}$ (Equation 14 in Box 2). Our framework therefore indicates that a sufficient resource supply is needed to enable predator persistence, leading to apparent competition, which weakens density dependence among prey. As a result, diversity peaks at intermediate productivity, giving rise to a unimodal productivity–diversity relationship.

### 4.3 The Emergence of Multiple PDRs Within One Framework

Thus far, we have used the control formula to explain the emergence of both increasing and unimodal PDRs. Density‐dependent mortality is a key mechanism for both, but whether an increasing or unimodal pattern emerges depends on the community structure, specifically the strength of apparent competition from a common predator. A structural shift between the two PDRs could thus result from ecological or evolutionary changes that lead to predator extinction, colonisation, or increasing predator specialisation. This suggests a testable hypothesis: in systems where predation pressure declines (e.g., through predator loss), diversity should shift to increase monotonically with productivity. We are not aware of any experimental study that directly tests this prediction, but this hypothesis could be tested with ecosystem manipulation experiments where predators are added or removed systematically.

More generally, our framework indicates that the strength of apparent competition from shared predation, not solely its presence or absence, has a strong control on the emergent PDR. We next illustrate the transition from the increasing to the unimodal PDR as a function of this control. We simulate ecosystems where species compete for a single limiting resource while varying the number of predator species (Figure 4a). We assume that each predator targets a distinct group of prey (Figure S5), with adjacent predators sometimes overlapping in prey species. When the number of predators is small (< 10 in our simulations), apparent competition from shared predation is strong, and the unimodal productivity–diversity curve emerges (Figure 4a). As the number of predators increases and their diets become more specialised, apparent competition is reduced, and the curve shifts towards an increasing pattern, eventually converging to the pattern seen in bottom‐up guilds where predation is entirely negligible. Thus, species‐specific predation (when the number of predators reaches 100 in our simulations) also generates an increasing PDR. Stronger density‐dependent mortality modulates the quantification of this curve and generally promotes higher species diversity at lower levels of productivity (Figure S6).

### 4.4 Insights Into Observed Patterns

Finally, we relate our framework and analysis of PDRs to observations. We analyse two plant community datasets that report contrasting PDRs (Figure 4, (Adler et al. 2011; Fraser et al. 2015)). We pool all data together across sites, with total biomass used as a proxy for productivity (Supporting Information). We find that statistically, the data from Adler et al. (2011) supports a significantly increasing PDR (the dashed line in Figure 4b), whereas the data from Fraser et al. (2015) exhibits a unimodal (hump‐shaped) relationship (the dashed line in Figure 4c). However, despite these differences, the patterns of the two datasets pooled across sites seem visually indistinguishable, highlighting the difficulty of discerning the PDR based on empirical data alone. Additionally, both original studies found that within‐site regressions reveal a diversity of patterns, which we also recovered, with increasing (‘positive linear’ in Figure 4b,c) and unimodal (‘concave‐down’) patterns as well as decreasing and concave‐up patterns, and that many sites indicated no significant relationship.

Our framework provides insight into these variable PDRs (Figure 4). For one, our simulations help to make it obvious that sampling only part of the productivity gradient can produce diverse PDR patterns (Figure 4a). For example, a decreasing relationship would be inferred if only the ‘right‐hand side’ component of the unimodal curve were observed. This subsampling could explain much of the within‐site PDR variability. Overall, our simulations suggest three general patterns. First, unimodal PDRs are most likely to emerge when productivity spans a wide range, because the persistence of a common predator requires a sufficient level of productivity. Resolving the unimodal curve empirically thus requires sampling across a sufficiently broad range, including sufficiently low, intermediate, and high levels of productivity. Second, increasing PDRs are more likely to be observed at low productivity levels in food webs, even with common predators (representing the ‘left‐hand side’ of a unimodal curve), as well as across a wide range in productivity in food webs with specialised predators or no predators. Third, declining PDRs tend to occur at relatively high productivity levels, where apparent competition is dominant throughout the entire range. In summary, we posit that density‐dependent mortality may be the mechanism underlying many emergent PDRs, with their differences controlled by the strength of apparent competition from shared predation.

## 5 Outlook

We have presented a quantitative framework for understanding how density‐dependent mortality shapes community structure and diversity. By extending resource competition theory, we quantified the specifics of how density dependence reshapes the boundary between competitive exclusion and coexistence, enlarging coexistence zones along resource gradients. Moving beyond graphical analyses of a few species, we derived a general control formula for two fundamental types of guilds within complex ecosystems: bottom‐up guilds, where species share a resource, and top‐down guilds, where species share a predator. This formula predicts species abundance distributions and attributes the abundance of any species to three mechanistic components: The average biomass set by the shared resource or predator, its deviation from the community‐averaged invasion growth rate, and the strength of density‐dependent mortality.

We provided a unifying explanation for the emergence of multiple diversity–productivity curves, depending on the food web details. We found that density‐dependent mortality is a mechanism that may underly PDRs, and we outlined the dynamics by which this mechanism may result in the emergence of the two most commonly observed forms—increasing and unimodal curves. This obviates the need to rely on environmental heterogeneity and other explanations as mechanisms (though they may also contribute). When predation is negligible or highly specialised, and thus apparent competition is negligible, increasing resource supply allows more species to persist, resulting in a monotonic increase in species richness with productivity. In contrast, when density is additionally constrained by shared predation, generating apparent competition, diversity peaks at intermediate productivity.

More generally, our results contribute to the necessary work of extending theoretical analysis of ecosystem structure to complex ecosystems, such as microbial communities with thousands of species. The control formula provides a tractable framework for predicting species abundances based on traits and measurable quantities, rather than phenomenological interaction coefficients used by the Lotka–Volterra equations. In this way, our mechanistic framework paves the way towards predictive capability in complex ecosystems.

## Author Contributions

L.X. and E.Z. conceived the study. C.A.K. helped refine the idea. L.X. performed the modelling work, theoretical derivation and simulations and analysed the simulated data. L.X., C.A.K. and E.Z. discussed biological interpretation. L.X. wrote the first draft of the manuscript, and all authors contributed substantially to revisions.

## Funding

This work was supported by the Simons Foundation and National Science Foundation, 2125142, EF‐2124800.

## Supporting information


**Data S1:** ele70292‐sup‐0001‐DataS1.pdf.

## Data Availability

All code and processed data supporting the results and generating the figures are archived in Zenodo (https://zenodo.org/records/17636644).
