# Supplementary material for: Density Dependence Promotes Species Coexistence and Provides a Unifying Explanation for Distinct Productivity–Diversity Relationships
Source: Ecol Lett. 2025 Dec 21;28(12):e70292. doi: 10.1111/ele.70292 (PMC12719913; doi:10.1111/ele.70292)
Supplement: Supplementary file 1 — Data S1: ele70292‐sup‐0001‐DataS1.pdf. [file ELE-28-0-s001.pdf]

Supplementary material: Density dependence promotes species coexistence and provides a unifying explanation for distinct productivity-diversity relationships

Liang Xu[1], Christopher A. Klausmeier[2] and Emily Zakem[1]
1. Department of Global Ecology, Carnegie Institution for Science, Stanford, CA, USA
2. W. K. Kellogg Biological Station, Department of Plant Biology & Integrative Biology, Program in Ecology, Evolution & Behavior, Michigan State University, USA

In this supplementary material, we first provide the analytical formulations under the assumptions of top-down and bottom-up guilds, as these serve as the foundation for deriving the boundaries of species coexistence zones in the resource space (Section 1). We then present the derivation of the boundaries defining the range of resource productivity that supports species coexistence in simplified guilds (Section 2). In Section 3, we derive the threshold of the resource supply rates that allowing a set of species to coexist under two guilds. We present standardized statistical analysis for the two global data sets in Section 4.

1 The ecological model and the control formulas

1.1 The bottom-up guild

The general model for the bottom-up guild, where consumer species are limited by a single resource, is defined as:

$$\frac{1}{N_i}\frac{dN_i}{dt} = \mu_i(R) - m_i - g_i(P_1, \cdots, P_h) - m_{qi}N_i^{x-1} \tag{1}$$

$$\frac{dR}{dt} = s(R) - \sum_i^n V_i(R)N_i. \tag{2}$$

At equilibrium, this system yields:

$$s(R) = \sum_i^n V_i(R)N_i \tag{3}$$

$$m_{qi}N_i^{x-1} = \mu_i(R) - m_i - g_i(P_1, \cdots, P_h). \tag{4}$$

Eqn.4 can be simplified by taking the root of $x-1$ on both sides, yielding

$$m'_{qi}N_i = G_i \tag{5}$$

where $m'_{qi} = \sqrt[x-1]{m_{qi}}, G_i = \sqrt[x-1]{\mu_i(R) - m_i - g_i(P_1, \cdots, P_h)}$. This formulation applies to any values of x.

A key approach with Eqn. 5 is to combine the equation for a focal species i with that of any other competitor j, yielding:

$$m'_{qi}N_i - m'_{qj}N_j = G_i - G_j,\ \text{for } j = 1, \cdots, n \text{ and } j \neq i. \tag{6}$$

Using the constraint condition Eqn. 3 and the contrast equation 6, we solve for N_i as:

$$N_i = \frac{s(R)}{\sum_j \frac{m'_{qi}}{m'_{qj}} V_j(R)} + \frac{1}{m'_{qi}} \left(G_i - \frac{\sum_j V_j(R) G_j / m'_{qj}}{\sum_j V_j(R)/m'_{qj}} \right) \tag{7}$$

or equivalently, emphasizing pairwise contrasts of G:

$$N_i = \frac{s(R)}{\sum_j \frac{m'_{qi}}{m'_{qj}} V_j(R)} + \frac{1}{m'_{qi}} \sum_k^n \frac{V_k(R)/m'_{qk}}{\sum_j V_j(R)/m'_{qj}} (G_i - G_k) \tag{8}$$

This highlights the pairwise contrasts of the net growth rates G_i and G_j, excluding the nonlinear mortality term, between the focal species and its competitors.

The special case presented in the main text (Eqns. 9 and 11) is derived by assuming that all consumer species share the same quadratic mortality rate $m_{qi} = m_{qj} = m_q$ and $x = 2$ corresponding to a quadratic mortality rate.

1.2 The top-down guild

The top-down guild, where consumer species are limited by a shared predator, is described by (Eqns. 10 in the main text):

$$\frac{1}{P}\frac{dP}{dt} = \sum_i f_i N_i - m_P \tag{9}$$

$$\frac{1}{N_i}\frac{dN_i}{dt} = \mu_i(R_1, \cdots, R_l) - m_i - g_i(P) - m_{qi}N_i^{x-1} \tag{10}$$

At equilibrium, the system satisfies:

$$\sum_i f_i N_i = m_P \tag{11}$$

$$m'_{qi}N_i - m'_{qj}N_j = G_i - G_j,\ \text{for } j = 1, \cdots, n \text{ and } j \neq i. \tag{12}$$

Eqn. 11 highlights how the shared predator's traits regulate the total density of consumer species. By eliminating the terms involving N_j, we obtain a general expression for top-down control:

$$N_i = \frac{m_P}{\sum_j \frac{m'_{qi}}{m'_{qj}} f_j} + \frac{1}{m'_{qi}} \left(\mu_{n,i} - \frac{\sum_j f_j G_j / m'_{qj}}{\sum_j f_j / m'_{qj}} \right), \tag{13}$$

or, in expanded form:

$$N_i = \frac{m_P}{\sum_j \frac{m'_{qi}}{m'_{qj}} f_j} + \frac{1}{m'_{qi}} \sum_k^n \frac{f_k/m'_{qk}}{\sum_j f_j/m'_{qj}} (G_i - G_k) . \tag{14}$$

When assuming a quadratic mortality rate $x = 2$ and equal quadratic mortality rates for all consumer species $m_{qi} = m_{qj} = m_q$, the top-down control formula simplifies to Eqn. 11 in the main text.

This derivation can also accommodate nonlinear predation response functions. For example, under a Holling Type II functional response:

$$f_i = \frac{a_i}{\sum_k a_k N_k + \kappa_P} \tag{15}$$

where a_i is the predation rate and κ_P is the half-saturation rate, we can solve Eqn. 11 and obtain

$$\sum_i a_i N_i = \frac{\kappa_P m_P}{1 - m_P}. \tag{16}$$

Subsequent derivation produces the top-down formula:

$$N_i = \frac{\frac{\kappa_P m_P}{1-m_P}}{\sum_j \frac{m'_{qi}}{m'_{qj}} a_j} + \frac{1}{m'_{qi}} \left(\mu_{n,i} - \frac{\sum_j a_j G_j / m'_{qj}}{\sum_j a_j / m'_{qj}} \right). \tag{17}$$

This modular framework can be broadly applied to various functional response forms, as long as equilibrium states can be expressed using linear equations, such as in the bottom-up guild (Eqns. 3 and 6) and the top-down guild (Eqns. 11 and 12).

2 The range of resource supply rates for species coexistence

2.1 Two generalists and two resources

The two-species, two-resources model describes the dynamics of two generalist species that each have unique advantages in utilizing different resources. The model equations are as follows:

$$\frac{1}{N_1}\frac{dN_1}{dt} = \mu_{11}R_1 + \mu_{21}R_2 - m - m_{q1}N_1 \tag{18}$$

$$\frac{1}{N_2}\frac{dN_2}{dt} = \mu_{12}R_1 + \mu_{22}R_2 - m - m_{q2}N_2 \tag{19}$$

$$\frac{dR_1}{dt} = a(s_1 - R_1) - V_{11}N_1R_1 - V_{12}N_2R_1 \tag{20}$$

$$\frac{dR_2}{dt} = a(s_2 - R_2) - V_{21}N_1R_2 - V_{22}N_2R_2. \tag{21}$$

Due to the symmetry in the dynamics of the two generalist species, we detail only the derivation for the case where Generalist 1 invades a community dominated by Generalist 2.

The critical equilibrium occurs when Generalist 2 is at equilibrium, and the invasion rate of Generalist 1 is zero. This condition is expressed as:

$$\mu_{11}R_1^* + \mu_{21}R_2^* = m \tag{22}$$

$$\mu_{12}R_1^* + \mu_{22}R_2^* - m = m_{q2}N_2^* \tag{23}$$

$$a(s_1 - R_1^*) = V_{12}N_2^*R_1^* \tag{24}$$

$$a(s_2 - R_2^*) = V_{22}N_2^*R_2^*. \tag{25}$$

Substituting Eqn. 23 to 24 and combining Eqn. 22 yield:

$$am_{q2}(s_1 - R_1^*) = V_{12}\mu_{12}R_1^{*2} + V_{12}\mu_{22}R_1^*(\frac{m}{\mu_{21}} - \frac{\mu_{11}}{\mu_{21}}R_1^*) - mV_{12}R_1^*. \tag{26}$$

We can solve for R_1^* as

$$R_1^* = \frac{-\left(mV_{12}(\mu_{22}-\mu_{21}) + am_{q2}\mu_{21}\right) - \sqrt{\left(mV_{12}(\mu_{22}-\mu_{21}) + am_{q2}\mu_{21}\right)^2 + 4am_{q2}s_1\mu_{21}V_{12}(\mu_{11}\mu_{22} - \mu_{12}\mu_{21})}}{2V_{12}(\mu_{12}\mu_{21} - \mu_{11}\mu_{22})} \tag{27}$$

The second solution of the quadratic equation is biologically irrelevant because it results in negative resource values.

From the bottom-up control formula on Resource 2 (Eqn. 7), at the critical equilibrium, the density of Generalist 1 is zero. This gives:

$$N_1^* = \frac{a(s_2 - R_2^*)}{R_2^*(V_{21} + V_{22})} + \frac{1}{m_{q1}}\frac{V_{22}}{V_{21} + V_{22}}\left((\mu_{11} - \mu_{12})R_1^* + (\mu_{21} - \mu_{22})R_2^*\right) = 0. \tag{28}$$

Solving for s_2, we find:

$$s_2 = R_2^* + \frac{R_2^*}{a}\left[\frac{V_{22}}{m_{q1}}\left((\mu_{11} - \mu_{12})R_1^* + (\mu_{21} - \mu_{22})R_2^*\right)\right]. \tag{29}$$

By substituting $R_2^* = (m - \mu_{11}R_1^*)/\mu_{21}$ and using the expression Eqn. 27, we can fully solve for s_2.

2.2 A third generalist invades a community of two generalists

Similarly, we can consider a model that examines the condition for one generalist invading a community of two generalists

$$\frac{1}{N_1}\frac{dN_1}{dt} = \mu_{11}R_1 + \mu_{21}R_2 - m - m_qN_1 \tag{30}$$

$$\frac{1}{N_2}\frac{dN_2}{dt} = \mu_{12}R_1 + \mu_{22}R_2 - m - m_qN_2 \tag{31}$$

$$\frac{1}{N_3}\frac{dN_3}{dt} = \mu_{13}R_1 + \mu_{23}R_2 - m - m_qN_3 \tag{32}$$

$$\frac{dR_1}{dt} = a(s_1 - R_1) - V_{11}N_1R_1 - V_{12}N_2R_1 - V_{13}N_3R_1 \tag{33}$$

$$\frac{dR_2}{dt} = a(s_2 - R_2) - V_{21}N_1R_2 - V_{22}N_2R_2 - V_{23}N_3R_2. \tag{34}$$

The equilibrium when the second generalist invades the community where other two generalists are at equilibrium, yielding:

$$\mu_{11}R_1^* + \mu_{21}R_2^* - m = m_q N_1^* \tag{35}$$

$$\mu_{12}R_1^* + \mu_{22}R_2^* - m = 0 \tag{36}$$

$$\mu_{13}R_1^* + \mu_{23}R_2^* - m = m_q N_3^* \tag{37}$$

$$a(s_1 - R_1^*) = V_{11}N_1^* R_1^* + V_{13}N_3^* R_1^* \tag{38}$$

$$a(s_2 - R_2^*) = V_{21}N_1^* R_2^* + V_{23}N_3^* R_2^*. \tag{39}$$

Substituting N_1^* and N_2^* solved from Eqn. 35 and 36, we have

$$\begin{aligned} &\left[V_{11}(\mu_{11}\mu_{22} - \mu_{12}\mu_{21}) + V_{13}(\mu_{13}\mu_{22} - \mu_{12}\mu_{23})\right] R_1^{*2} + \\ &\left[m(V_{11}(\mu_{21} - \mu_{22}) + V_{13}(\mu_{23} - \mu_{22})) + m_q\mu_{22}\right] R_1^* - m_q s_1 \mu_{22} = 0 \end{aligned} \tag{40}$$

which gives:

$$R_1^* = \frac{-\left[m(V_{11}(\mu_{21} - \mu_{22}) + V_{13}(\mu_{23} - \mu_{22})) + m_q\mu_{22}\right]}{2\left[V_{11}(\mu_{11}\mu_{22} - \mu_{12}\mu_{21}) + V_{13}(\mu_{13}\mu_{22} - \mu_{12}\mu_{23})\right]} + \tag{41}$$

$$\frac{\sqrt{\left[m(V_{11}(\mu_{21} - \mu_{22}) + V_{13}(\mu_{23} - \mu_{22})) + m_q\mu_{22}\right]^2 + 4m_q s_1 \mu_{22}\left[V_{11}(\mu_{11}\mu_{22} - \mu_{12}\mu_{21}) + V_{13}(\mu_{13}\mu_{22} - \mu_{12}\mu_{23})\right]}}{2\left[V_{11}(\mu_{11}\mu_{22} - \mu_{12}\mu_{21}) + V_{13}(\mu_{13}\mu_{22} - \mu_{12}\mu_{23})\right]} \tag{42}$$

We can use Eqn. 36 to solve R_2^*. By applying the bottom-up formulas on both resources, one can get the analytical solution for the boundaries for the three species to coexist. Fig. S7 demonstrates how the density-dependent mortality changes the boundaries of the pairwise coexistence regions and makes them merge at a high supply vector.

2.3 A general solution to R_i^*

We generalize the approach to derive the equilibrium density of Resource 1, when a Generalist i invades a community of many other generalists $1, \cdots, j$. The governing equation for R_1^* is:

$$R_1^* = \frac{-\left[m\sum_j V_{1j}(\mu_{2j} - \mu_{2i}) + m_q\mu_{2i}\right] + \sqrt{\left[m\sum_j V_{1j}(\mu_{2j} - \mu_{2i}) + m_q\mu_{2i}\right]^2 + 4m_q s_1 \mu_{2i}\sum_j V_{1j}(\mu_{1j}\mu_{2i} - \mu_{1i}\mu_{2j})}}{2\sum_j V_{1j}(\mu_{1j}\mu_{2i} - \mu_{1i}\mu_{2j})}. \tag{43}$$

Together with the equilibrium state of the invader, we can solve the boundaries for species coexistence.

2.4 Two specialists, one generalist and two resources

We consider a simplified model comprising two specialist species and one generalist species competing for two resources, as an illustrative example for the case involving three generalists. This setup allows for an explicit analytical derivation of coexistence boundaries and facilitates straightforward graphical interpretation in the main text.

In this model, specialists (species 1 and 2) exclusively consume resource 1 and resource 2, respectively, while the generalist (species 3) can utilize both resources. Figure 2h shows the Zero-Net-Growth Isoclines (ZNGIs) of the three species intersecting at distinct points, demonstrating that in the absence of density-dependent effects, at most two species can coexist.

When density dependence is incorporated, mutual invasion conditions can be derived using the framework previously outlined. A key consideration for three-species coexistence is that the coexistence boundary must be evaluated by examining invasion scenarios in which a third species attempts to invade a community already containing the other two.We focus on the invasion dynamics of a specialist attempting to invade a resident community composed of the generalist and the other specialist.

The critical equilibrium conditions are given by:

$$\mu_{11}R_1^* = m \tag{44}$$
$$\mu_{22}R_2^* - m = m_q N_2^* \tag{45}$$
$$\mu_{13}R_1^* + \mu_{23}R_2^* - m = m_q N_3^* \tag{46}$$
$$a(s_1 - R_1^*) = V_{13}N_3^*R_1^* \tag{47}$$
$$a(s_2 - R_2^*) = V_{22}N_2^*R_2^* + V_{23}N_3^*R_2^*. \tag{48}$$

Substituting N_3^* in Eqn. 47 yields

$$a(s_1 - R_1^*) = \frac{V_{13}R_2^*}{m_q}(\mu_{13}R_1^* + \mu_{23}R_2^* - m). \tag{49}$$

Given $R_1^* = m/\mu_{11}$, we can solve for R_2^* as:

$$R_2^* = \frac{-\left(mV_{13}(\mu_{13} - \mu_{11}) + m_q a\mu_{11}\right) + \sqrt{\left(mV_{13}(\mu_{13} - \mu_{11}) + m_q a\mu_{11}\right)^2 + 4m_q a s_1 \mu_{11}^2 \mu_{23} V_{13}}}{2V_{13}\mu_{11}\mu_{23}}. \tag{50}$$

From Eqn. 48, s_2 can be expressed as:

$$s_2 = R_2^* + \frac{1}{a}\left(V_{22}N_2^*R_2^* + V_{23}N_3^*R_2^*\right) \tag{51}$$

Together with Eqns. 45 and 46 and substituting R_1^* and R_2^*, we can obtain the full solution for the new boundary. The full solution for the coexistence boundary can be obtained. The resulting coexistence curve is illustrated in Figure 2 of the main text.

2.5 Two consumer species, one predator and one resource

The diamond-shaped food web can be described by the following system of equations:

$$\frac{1}{P}\frac{dP}{dt} = f_1N_1 + f_2N_2 - m_p \tag{52}$$
$$\frac{1}{N_1}\frac{dN_1}{dt} = \mu_1 R - m - g_1 P - m_q N_1 \tag{53}$$
$$\frac{1}{N_2}\frac{dN_2}{dt} = \mu_2 R - m - g_2 P - m_q N_2 \tag{54}$$
$$\frac{dR}{dt} = a(s - R) - V_1N_1R - V_2N_2R. \tag{55}$$

Because of symmetry, we derive the invasion condition for one species (species 1) invading a community dominated by the other (species 2).

To examine the critical equilibrium where species 1 is on the verge of invasion while species 2 dominates, we set $N_1^* \to 0$ and the equilibrium conditions are:

$$\begin{aligned} f_2 N_2 - m_p &= 0 \\ \mu_1 R - m - g_1 P &= 0 \\ \mu_2 R - m - g_2 P - m_q N_2 &= 0 \\ a(s - R) - V_2 N_2 R &= 0 \end{aligned}$$

Solving these equations for R , the equilibrium resource availability when species 1 invades can be expressed as:

$$R = \frac{m(g_1 - g_2) - m_q \frac{m_p}{f_2} g_1}{g_1 \mu_2 - g_2 \mu_1} \tag{56}$$

The resource productivity threshold for the invasion of species 1 $s_{12}$is given by:

$$s_{12} = \frac{(g_1 - g_2)m + m_q m_p \frac{g_1}{f_2}}{g_1 \mu_2 - g_2 \mu_1} (1 + \frac{m_p}{a} \frac{V_2}{f_2}) \tag{57}$$

where the superscript on s_{12} denotes the invasion of Species 1.

Similarly, when species 2 invades a community dominated by species 1, the resource productivity threshold (s_{21}) can be expressed as:

$$s_{21} = \frac{(g_1 - g_2)m - m_q m_p \frac{g_2}{f_1}}{g_1 \mu_2 - g_2 \mu_1} (1 + \frac{m_p}{a} \frac{V_1}{f_1}) \tag{58}$$

The interval of resource productivity where both species coexist is expanded by self-limitation, as evident from the separation between s_{21} and s_{12}. This demonstrates how self-regulation mechanisms influence species coexistence dynamics in the diamond food web.

Note that previous findings of the diamond food web in the absence of density dependence ($m_q = 0$) is a special case. The coexistence of two species can only occur when the ZNGIs intersect. From Eqns. 57 and 58, two critical resource supply rates are given by $s_{21} = \frac{(g_1 - g_2)m}{g_1\mu_2 - g_2\mu_1}\left(1 + \frac{V_1 m_p}{a f_1}\right)$ and $s_{12} = \frac{(g_1 - g_2)m}{g_1\mu_2 - g_2\mu_1}\left(1 + \frac{V_2 m_p}{a f_2}\right)$ where s_{ij} denotes the threshold of the supply rate for species i invading species j. We see that coexistence of the two species requires $\frac{V_1}{f_1} < \frac{V_2}{f_2}$ and $\frac{\mu_1}{g_1} < \frac{\mu_2}{g_2}$, which refers to that the condition that better resource-competitor (species 1) has a steeper resource-predator impact vector than the better defended (species 2) (Leibold 1996; Chase and Leibold 2003). Otherwise, we have $s_{21} > s_{12}$, where a priority effect emerges in the interval of $(s_{12},\ s_{21})$.

2.6 Three consumer species, one predator and one resource

We examine the resource productivity threshold at which species 3 can invade a community already consisting of species 1 and species 2. At equilibrium, the system is described by the following equations:

$$f_1 N_1 + f_2 N_2 - m_p = 0 \tag{59}$$

$$\mu_1 R - m - g_1 P = m_q N_1 \tag{60}$$

$$\mu_2 R - m - g_2 P = m_q N_2 \tag{61}$$

$$\mu_3 R - m - g_3 P = 0 \tag{62}$$

$$a(s - R) = V_1 N_1 R + V_2 N_2 R. \tag{63}$$

In this diamond food web, all three species share a common predator and resource, allowing for the application of both bottom-up and top-down approaches. Without loss of generality, we assume: $\mu_1 > \mu_2 > \mu_3$ and $g_1 > g_2 > g_3$. Using the top-down perspective, the equilibrium density of species 3 can be expressed as:

$$N_3^* = \frac{m_P}{f_1 + f_2 + f_3} + \frac{1}{m_q} \frac{f_1}{f_1 + f_2 + f_3} (G_3 - G_1) + \frac{1}{m_q} \frac{f_2}{f_1 + f_2 + f_3} (G_3 - G_2)\,. \tag{64}$$

As $N_3^* \to 0$, together with Eqn. 62, we have

$$R = \frac{m(f_1 + f_2) - (f_1 g_1 + f_2 g_2)\frac{m}{g_3} + m_q m_p}{(f_1\mu_1 + f_2\mu_2) - (f_1 g_1 + f_2 g_2)\frac{\mu_3}{g_3}}. \tag{65}$$

Similarly, using the bottom-up perspective, the equilibrium density of species 3 can also be expressed as:

$$N_3^* = \frac{a(s-R)}{V_1 + V_2 + V_3} + \frac{1}{m_q}\frac{V_1}{V_1 + V_2 + V_3}(G_3 - G_1) + \frac{1}{m_q}\frac{V_2}{V_1 + V_2 + V_3}(G_3 - G_2). \tag{66}$$

Again, substituting $N_3^* \to 0$ and $P = \frac{\mu_3}{g_3}R - \frac{m}{g_3}$, we obtain the resource productivity threshold:

$$s = \frac{(V_1\mu_1 + V_2\mu_2) - (V_1 g_1 + V_2 g_2)\frac{\mu_3}{g_3}}{am_q}R^2 + \frac{m\left(\frac{V_1 g_1 + V_2 g_2}{g_3} - (V_1 + V_2)\right)}{am_q}R + R. \tag{67}$$

Replacing R with its expression derived from the equilibrium equations 65 provides the analytical solution for the resource productivity threshold at which species 3 can invade a community dominated by species 1 and species 2.

3 The threshold of the supply rate when many species coexist on one resource

3.1 The fitness G increases with the supply rate in a bottom-up guild

Here, we show that the species fitness G (the net growth rate) can be an increasing, a constant and a decreasing function of the supply rate, depending on the structure of the guild. We first consider a bottom-up guild without predators. The dynamics of the prey and the resource are given by

$$\frac{1}{N_i}\frac{dN_i}{dt} = \mu_i R - m_i - m_q N_i \tag{68}$$

$$\frac{dR}{dt} = a(s-R) - \sum_i^n V_i R N_i. \tag{69}$$

where $G_i = \mu_i R - m_i$. At equilibrium we have the density given by

$$N_i = \frac{\mu_i R - m_i}{m_q}. \tag{70}$$

Substituting Eqn. 70 to 69 yields

$$a(s-R) = \sum_{i=1}^n V_i(\frac{\mu_i R - m_i}{m_q})R \tag{71}$$

which can be simplified to

$$a(s-R) = \frac{R}{m_q}[R\sum_i V_i\mu_i - m\sum_i V_i]. \tag{72}$$

Thus, we obtain

$$R = \frac{mB - am_q + \sqrt{(am_q - mB)^2 + 4Aam_q s}}{2A} \tag{73}$$

where $A = \sum_{i=1}^n V_i\mu_i, B = \sum_{i=1}^n V_i$. Eqn. 73 indicates that R is a monotonically increasing function of the supply rate s

$$\frac{dR}{ds} = \frac{am_q}{\sqrt{4Aam_q s + (mB - am_q)^2}} > 0 \tag{74}$$

. Thus, in this guild, the net growth rate G_i is also a monotonically increasing function of the supply rate s according to the derivatives

$$\frac{dG_i}{ds} = \mu_i\frac{dR}{ds} > 0 \tag{75}$$

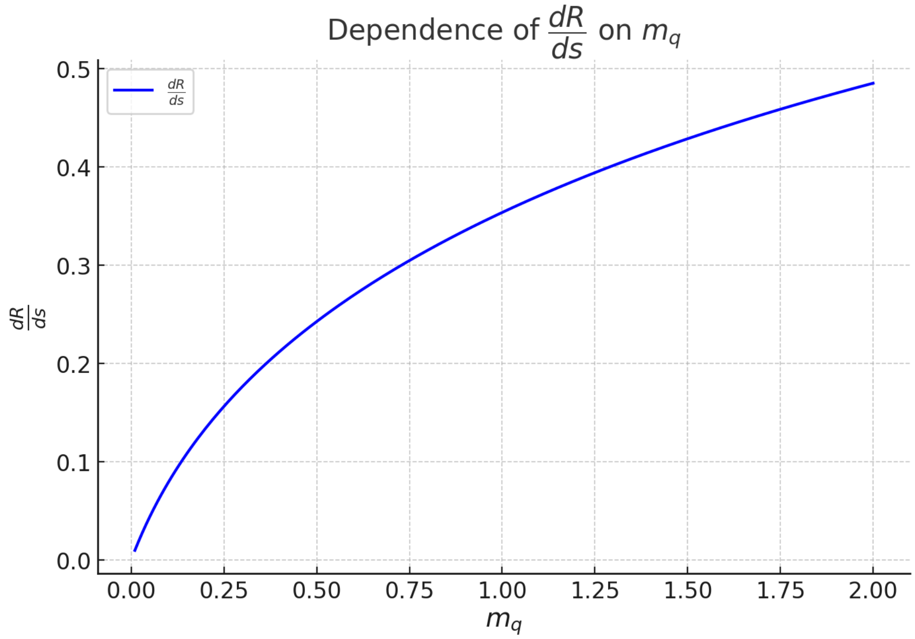

Fig.S 1: The derivative $\frac{dR}{ds}$ as a function of the density-dependent mortality rate m_q.

3.2 The fitness G first increases and then decreases with the supply rate in a diamond guild

Now we consider a diamond guild prey and a shared predator, given by

$$\frac{1}{P}\frac{dP}{dt} = \sum_i f_i N_i - m_P \tag{76}$$

$$\frac{1}{N_i}\frac{dN_i}{dt} = \mu_i R - m_i - g_i P - m_q N_i \tag{77}$$

$$\frac{dR}{dt} = a(s-R) - \sum_i^n V_i R N_i \tag{78}$$

where $G_i = \mu_i R - m_i - g_i P$. The density of prey species at equilibrium is then balanced between the predator and the resource, yielding

$$N_i = \frac{\mu_i R - m_i - g_i P}{m_q}. \tag{79}$$

Then, the resource equation (Eqn. 77) at equilibrium becomes

$$a m_q (s-R) = R(AR - mB - CP) \tag{80}$$

where $A = \sum_{i=1}^n V_i \mu_i, B = \sum_{i=1}^n V_i, C = \sum_{i=1}^n V_i g_i$.

Similarly, the density of the shared predatory can be solved as

$$P = \frac{DR - mE - m_q m_p}{F} \tag{81}$$

where $D = \sum_i f_i \mu_i, E = \sum_i f_i, F = \sum_i f_i g_i$. The two equations (Eqn. 80 and 81) together yield the solution for R at equilibrium

$$R = \frac{-\beta + \sqrt{\beta^2 + 4\alpha\gamma}}{2\alpha} \tag{82}$$

where $\alpha = A - \frac{CD}{F}, \beta = \frac{CmE}{F} + \frac{Cm_q m_p}{F} - mB + am_q, \gamma = am_q s$.

We want to know how G_i changes with the supply rate s. We can take the derivative of G_i with respect of s, yielding

$$\frac{dG_i}{ds} = \mu_i \frac{dR}{ds} - g_i \frac{dP}{ds}. \tag{83}$$

$\frac{dP}{ds}$ can be expressed as a function of $\frac{dR}{ds}$. From Eqn. 81, taking the derivative with respect to s yields

$$\frac{dP}{ds} = \frac{\sum_i f_i \mu_i}{\sum_i f_i g_i} \frac{dR}{ds}. \tag{84}$$

Then, the final expression for $\frac{dG_i}{ds}$ is

$$\frac{dG_i}{ds} = (\mu_i - g_i \frac{\sum_j f_j \mu_j}{\sum_j f_j g_j}) \frac{dR}{ds}. \tag{85}$$

This expression makes it clear that the sign of $\frac{dG_i}{ds}$ depends on how much $\mu_i$exceeds the predator feedback term and that $\frac{dR}{ds} > 0$ under density dependence. For the latter, Fig. S3 shows a positive relationship between the resource concentration at equilibrium and the supply rate for communities with predators.

Here, we prove that $(\mu_i - g_i \frac{\sum_j f_j \mu_j}{\sum_j f_j g_j})$ decreases with add more species from the index i with lower μ_j, g_j. We define $T_{j-i} := \mu_i - g_i \frac{\sum_j f_j \mu_j}{\sum_j f_j g_j}$ decreases with adding $\mu_j, g_j, j > i$, where $j - i$ indicates diversity. As mentioned above, we consider a sequence of invasion, i.e., $\frac{\mu_1}{g_1} < \cdots < \frac{\mu_i}{g_i}$. Thus, we can write

$$\frac{\mu_i}{g_i} = \alpha_i < \cdots < \frac{\mu_j}{g_j} = \alpha_j. \tag{86}$$

For two species, we have

$$T_2 = \mu_i - g_i \frac{f_i \mu_i + f_{i+1} \mu_{i+1}}{f_i g_i + f_{i+1} g_{i+1}} = \mu_i - g_i \frac{f_i \alpha_i g_i + f_{i+1} \alpha_{i+1} g_{i+1}}{f_i g_i + f_{i+1} g_{i+1}} < \mu_i - g_i \frac{f_i \alpha_i g_i + f_{i+1} \alpha_i g_{i+1}}{f_i g_i + f_{i+1} g_{i+1}} = \mu_i - g_i \alpha_i = T_1 \tag{87}$$

We can also prove that when species composition shifts from the first species to the i-th species, $\mu_i - g_i \frac{\sum_{j=k}^{i} f_j \mu_j}{\sum_{j=k}^{i} f_j g_j}$ is positive. In fact, we have

$$\mu_i - g_i \frac{f_{i-1} \mu_{i-1} + f_i \mu_i}{f_{i-1} g_{i-1} + f_i g_i} > \mu_i - g_i \frac{f_i \mu_i}{f_i g_i} = 0 > \mu_i - g_i \frac{f_{i+1} \mu_{i+1} + f_i \mu_i}{f_{i+1} g_{i+1} + f_i g_i} \tag{88}$$

Thus, $\frac{dG_i}{ds}$ decreases from positive to negative values with the increase of the supply s.

Note that When only a single species occupies the diamond-shaped trophic guild, the derivative $\frac{dG_i}{ds} = 0$, implying that the density of that species is independent of the supply rate. In this scenario, prey density remains constant and is determined solely by the predator's trait. Specifically, from Eqn. 85, we have $N_i = \frac{m_p}{f_i}$ when only a single prey species is present.

3.3 The criteria of the bottom-up guild

Consider a community of multiple species competing for a single resource. The dynamics of the species and the resource are governed by the following differential equations:

$$\frac{1}{N_i} \frac{dN_i}{dt} = \mu_i R - m - m_q N_i \tag{89}$$

$$\frac{dR}{dt} = a(s - R) - \sum_j V_j N_j R. \tag{90}$$

Let's order the species by their growth rates μ_i, assuming that $\mu_1 > \mu_2 > \cdots > \mu_i$. Thus, species i is considered the most inferior species in terms of its growth rate. If species i can persist in this system, all species can coexist. Using the bottom-up formula (Eqn. 7), the equilibrium density of species i is given by:

$$N_i^* = \frac{a(s - R^*)}{\sum_j V_j R^*} + \frac{1}{m_q}\left(G_i - \bar{G}_{BU}\right) > 0 \tag{91}$$

$$R^* > \frac{m}{\mu_i}. \tag{92}$$

The conditions derived above lead to a threshold for the supply rate of the resource s that can sustain species i. This threshold is given by:

$$s > \frac{m}{\mu_i} + \frac{1}{am_q}\sum_{j=1}^{i-1} V_j(\mu_j - \mu_i)(\frac{m}{\mu_i})^2 \tag{93}$$

which is Eqn. 10 in the main text.

3.4 The criteria of the diamond food web

The key implication of the control formula is that a given set of species can coexist as long as the species with the lowest growth rate (G) has a non-negative value for its G. In a diamond-shaped food web where multiple species occupy intermediate trophic levels, the model is described by the following system of equations:

$$\frac{1}{P}\frac{dP}{dt} = \sum_h f_h N_h - m_p \tag{94}$$

$$\frac{1}{N_i}\frac{dN_i}{dt} = \mu_i R - m - g_i P - m_q N_i \tag{95}$$

$$\frac{dR}{dt} = s - R - \sum_j V_j N_j R. \tag{96}$$

We assume that species are ranked according to their growth rates, from highest to lowest, and similarly by their predation rates g_i from highest to lowest. To allow for pairwise coexistence without the effects of negative density dependence, we impose the condition:

$$\frac{\mu_1}{g_1} < \cdots < \frac{\mu_i}{g_i}. \tag{97}$$

As shown in Section 2, the negative density dependence expands the interval of pairwise coexistence on the resource axis, thereby allowing more species to coexist. In such an interval of resource concentration where a set of species coexist, we must identify the species with the lowest value of Let's assume that species $(j = i, \cdots, k)$ can coexist in the interval (R_s, R_e). There are two possibilities for which species has the lowest G.

1. The species with the largest growth and predation rates: In this case, the species with the largest growth and predation rates must first be excluded due to apparent competition as the resource concentration increases within the interval. However, in the second part of the interval, the species with the largest growth and predation rates will have the lowest G.

2. The species with the lowest growth and predation rates: This species is the first to be excluded as the resource concentration decreases within the interval, since it is close to the point of invasion by the other species in the cohort. In the first part of the interval (R_s, R_m), the species with the lowest growth and predation rates will have the lowest G. As the supply rate increases, the values of G for these two species will converge at R_m, where both species have the same lowest G.

Let's assume a middle point R_m that in the interval (R_m, R_e) the species of the largest growth rate and predation rate has lowest G. Therefore, in this interval, we have the minimum requirement for

$$G_j > G_i \text{ for } j \neq 1 \tag{98}$$
$$G_i = 0 \tag{99}$$
$$N_i > 0 \tag{100}$$

From Eqn. 100 and the bottom-up control formula, we have

$$N_i = \frac{(s - R^*)}{\sum_j V_j R^*} + \frac{1}{m_q} \frac{\sum_j V_j (G_i - G_j)}{\sum_j V_j} > 0 \tag{101}$$

which leads to

$$s > \frac{1}{m_q} \sum_j V_j R^* \left[(\mu_j - \mu_i) R^* - (g_j - g_i) P^* \right] + R^*. \tag{102}$$

From the condition Eqn. 99, we know that $P^* = (\mu_i R^* - m)/g_i$ and insert it into Eqn. 102, we have

$$s > \frac{1}{m_q} \sum_j V_j (\mu_j - \frac{g_j}{g_i} \mu_i) R^{*2} + \frac{1}{m_q} \sum_j V_j (g_j - g_i) \frac{m}{g_i} R^* + R^*. \tag{103}$$

The inequality Eqn. 103 predicts that species can coexist if the supply rate exceeds this threshold. The right-hand term is a quadratic curve opening upwards because $\mu_j - \frac{g_j}{g_i}\mu_i > 0$ given the ranking assumption in equation 97.

Conversely, the species with the lowest growth and predation rates will be excluded first as resource concentration decreases. In the first part of the interval (R_s, R_m), the species with the lowest growth and predation rates has the lowest G. As the supply rate increases, the values of G for the two species will converge at R_m. Similarly, we derive the condition for the supply rate in the first part of the interval where coexistence is possible:

$$s > \frac{1}{m_q} \sum_j V_j (\mu_j - \frac{g_j}{g_k} \mu_k) R^{*2} + \frac{1}{m_q} \sum_j V_j (g_j - g_k) \frac{m}{g_k} R^* + R^*. \tag{104}$$

In this case, the right-hand term is a quadratic curve opening downward since $\mu_j - \frac{g_j}{g_k}\mu_k < 0$.

Now, we determine the value of R_m. Because at this point both kinds of species have the same G and the minimum requirement is $G = 0$. Thus, we have

$$G_k = G_i = 0 \tag{105}$$

which leads to

$$R_m = \frac{m(\frac{1}{g_k} - \frac{1}{g_i})}{(\frac{\mu_k}{g_k} - \frac{\mu_i}{g_i})}. \tag{106}$$

It is clear that R_m exists since both the numerator and denominator are positive.

Thus, the condition for the supply rate that allows for species coexistence is:

$$s > \begin{cases} \frac{1}{m_q} \sum_j V_j (\mu_j - \frac{g_j}{g_k} \mu_k) R^{*2} + \frac{1}{m_q} \sum_j V_j (g_j - g_k) \frac{m}{g_k} R^* + R^* & R^* \in (R_s, R_m) \\ \frac{1}{m_q} \sum_j V_j (\mu_j - \frac{g_j}{g_i} \mu_i) R^{*2} + \frac{1}{m_q} \sum_j V_j (g_j - g_i) \frac{m}{g_i} R^* + R^* & R^* \in (R_m, R_e) \end{cases} \tag{107}$$

$$R_m = \frac{m(\frac{1}{g_k} - \frac{1}{g_i})}{(\frac{\mu_k}{g_k} - \frac{\mu_i}{g_i})} \tag{108}$$

when species are ranked as

$$\frac{\mu_i}{g_i} < \cdots < \frac{\mu_k}{g_k}$$

This condition provides a mechanistic and analytical explanation for the emergence of unimodal or multimodal curves in species coexistence.

1. **Curve Characteristics**: In the first part of the interval (Eqn. 107), the curve is a downward-opening quadratic curve because $\mu_j - \frac{g_j}{g_k}\mu_k < 0$ for all $j < k$ based on our ordering assumption. Conversely, in the second part of the interval (Eqn. 108), the curve is an upward-opening quadratic curve because $\mu_j - \frac{g_j}{g_i}\mu_i > 0$ for all $j > i$. Together, these segments form a shape illustrated in Fig.4. This curve characteristics predicts that the coexistence of many species can only achieved at an intermediate concentration of resource.

2. **Effect of Negative Density Dependence**: Negative density dependence decreases the coefficient of the quadratic term, thereby widening the curve's opening. This reduces the minimum supply rate requirement.

3. **General Applicability**: Unimodality is one specific outcome of a more general outcome: This criterion can be applied to any set of species to determine whether unimodality or multimodality emerge. To illustrate this, in the supplement we provide examples demonstrating the application of these criteria (See Fig.4).

Figure 4 illustrates three examples of the diamond food web, with the number of species being 2, 3, and 4. Panels (a), (b), (c), and (d) in Figure 4 display the criteria curves for species coexistence. Species coexistence is achieved when the supply-resource curve lies above the criteria curve. With the influence of density dependence, the diversity-productivity curve exhibits a unimodal shape. In contrast, when the effect of negative density dependence is weak or absent and the actual $S - \hat{R}$ curve is below the $s_{(min,123)} - \hat{R}$ curve for the coexistence of species 1, 2 and 3 but above the $s_{(min,12)} - \hat{R}, s_{(min,23)} - \hat{R}$, $s_{(min,34)} - \hat{R}$ curves for pairwise coexistence, a multimodal curve is produced (Fig. 4 (c), (g)), where the coexistence of one species pair is replaced by another along the resource gradient.

3.5 The criteria of the bottom-up guild with prey-specific predator

Consider a community of multiple consumer species competing for a single resource. Each consumer species N_i has its own prey-specific predator P_i. The system dynamics are described by:

$$\frac{1}{P_i}\frac{dP_i}{dt} = f_i N_i - m_p \tag{109}$$

$$\frac{1}{N_i}\frac{dN_i}{dt} = \mu_i R - m - g_i P_i - m_q N_i \tag{110}$$

$$\frac{dR}{dt} = a(s - R) - \sum_j V_j N_j R. \tag{111}$$

From Eqn. 109, predator persistence requires a minimum consumer density of,

$$N_i^* = m_p / f_i. \tag{112}$$

Therefore, each prey-specific predator can at most cap the density of its consumer species but cannot drive it extinct. Biologically, this means that when predators are strictly species-specific, they do not contribute to diversity loss among consumer species. Once a consumer species is established, its predator can invade and persist, drawing additional energy from the system without displacing the prey.

When the predator is present, increases in the external resource supply rate (s) primarily enhance predator density rather than prey density. The consumed resource flux is thus transferred from prey biomass to predator biomass, while prey abundance remains approximately constant. To formalize it, we combine Eqn. 109 and 111

$$\frac{m_q}{f_i}\frac{1}{P_i}\frac{dP_i}{dt} + \frac{1}{N_i}\frac{dN_i}{dt} = \mu_i R - m - g_i P_i - m_p \frac{m_q}{f_i}. \tag{113}$$

Equation 113 describes the general time-dependent interaction between prey and its predator. To gain analytical insight, we next consider the **quasi-equilibrium state** of the prey population, where prey dynamics are much faster than those of the predator and thus relax rapidly to a steady state $\frac{dN_i}{dt} = 0$. Under this assumption, the dynamics of predator becomes

$$\frac{1}{P_i}\frac{dP_i}{dt} = \frac{f_i}{m_q}\mu_i R - (m\frac{f_i}{m_q} + m_p) - g_i \frac{f_i}{m_q} P_i. \tag{114}$$

This quasi-equilibrium approximation is commonly used in consumer–resource models when predator dynamics are slower than prey or resource dynamics (e.g., analogous to a reduced Lotka–Volterra system). Mathematically, Eqn. 114 is equivalent in form to the consumer dynamics in a pure bottom-up system without predators, implying that the inclusion of species-specific predators does not alter the overall structure of the productivity–diversity relationship.

The only difference is the criterion of the supply rate. Again, let's order the species by their growth rates μ_i, assuming that $\mu_1 > \mu_2 > \cdots > \mu_i$. Thus, species i is considered the most inferior species in terms of its growth rate. If species i can persist in this system, all species can coexist. Using the bottom-up formula (Eqn. 7), the equilibrium density of species i is given by:

$$N_i^* = \frac{a(s - R^*)}{\sum_j V_j R^*} + \frac{1}{m_q}\left(G_i - \bar{G}_{BU}\right) > 0 \tag{115}$$

$$R^* > \frac{m}{\mu_i}. \tag{116}$$

The conditions derived above lead to a threshold for the supply rate of the resource s that can sustain species i. At this critical point when species i is just able to establish, its predator P_i cannot exist, thus, $P_i = 0$. This threshold is given by:

$$s > \frac{m}{\mu_i} + \frac{1}{am_q}\sum_{j=1}^{i-1} V_j(\mu_j - \mu_i)(\frac{m}{\mu_i})^2 - \frac{1}{am_q}\sum_{j=1}^{i-1} V_j g_j P_j \tag{117}$$

This formula indicates that the prey-specific predator generally decreases the requirement of productivity that allows species i to invade. Because with the predator the resource concentration achieves a higher value $R^* = (m + g_i P_i)/\mu_i$ even without density dependence.

4 The analysis of two global data sets

The two data sets are fetched from Adler et al. 2011(Adler et al. 2011) and Fraser et al. 2015 (Fraser et al. 2015). Both sampled data from a set of sites globally. We followed the statistical analysis as described in Adler et al. 2011, which is similar to the methods used by Fraser et al. 2015. The biomass is log10 transformed. We fit a model in which species richness was a linear and quadratic function of live biomass. If the quadratic term was not significant at the α=0.1 level, we dropped the quadratic term and fit a model containing only the linear term. If the linear term was not significant at α=0.1, we classified the relationship as nonsignificant. If the quadratic function and the linear function are both significant, we classified the relationship as quadratic. This approach, which is consistent with previous meta-analyses, emphasizes hypothesis testing of the quadratic term rather than overall model fit. The two regression functions are as followed:

$$\text{Richness} = a_0 + a_1 log10(\text{Biomass}) + a_2 log10(\text{Biomass})^2$$
$$\text{Richness} = a_0 + a_1 log10(\text{Biomass})$$

The statistic significant is given by the table below:

Data	Type of regression	Intercept estimate	Linear term coefficient	Quadratic term coefficient
Adler et al. 2011	quadratic	1.55942	0.61778 ($p < 0.137660$)	-0.07887 ($p < 0.391939$)
	linear	1.93511	0.26948 ($p < 2.23e^{-9}$)	NA
Fraser et al. 2015	quadratic	-2.53752	4.70012 ($p < 2e^{-16}$)	-1.04040 ($p < 2e^{-16}$)
	linear	3.91329	-0.52439 ($p < 2e^{-16}$)	NA

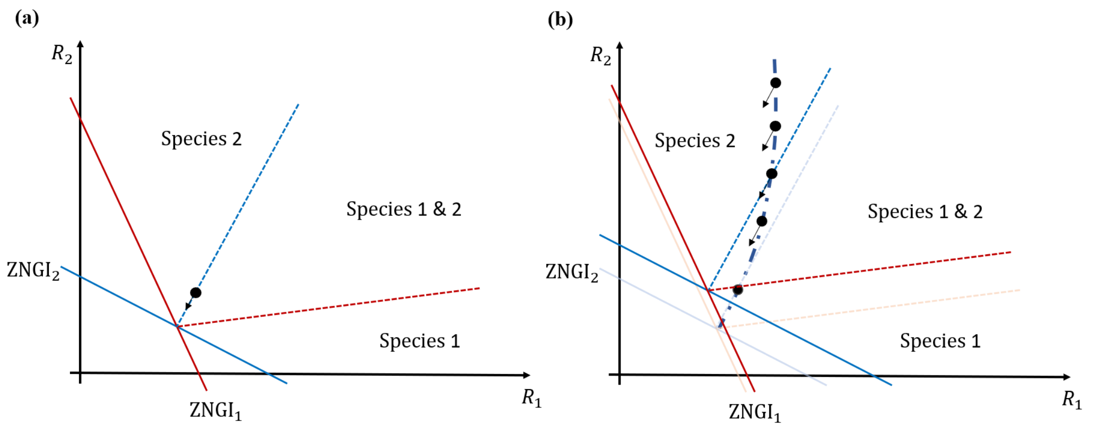

Fig.S 2: Illustration of how density-dependent effect changes the boundary between species dominance and species coexistence zones. The black dots denote the supply rates. (a) Without density-dependent effects: Species coexistence is limited to the supply rates that fall within the coexistence zone. The two species can only coexist at the point where their original Zero Net Growth Isoclines (ZNGIs) intersect; (b) With density-dependent effects: Increasing supply rates shifts the ZNGIs upwards, allowing the species to coexist at new points that were previously outside the original coexistence zone. This shift enables coexistence at a wider range of supply rates where it is not possible before.

References

Adler, Peter B. et al. (2011). "Productivity Is a Poor Predictor of Plant Species Richness". In: *Science* 333.6050, pp. 1750–1753. DOI: doi:10.1126/science.1204498. URL: https://www.science.org/doi/abs/10.1126/science.1204498.

Chase, Jonathan M. and Mathew A. Leibold (2003). *Ecological Niches: Linking Classical and Contemporary Approaches*. University of Chicago Press. ISBN: 9780226101798. DOI: 10.7208/chicago/9780226101811.001.0001. URL: https://doi.org/10.7208/chicago/9780226101811.001.0001.

Fraser, Lauchlan H. et al. (2015). "Worldwide evidence of a unimodal relationship between productivity and plant species richness". In: *Science* 349.6245, pp. 302–305. DOI: doi:10.1126/science.aab3916. URL: https://www.science.org/doi/abs/10.1126/science.aab3916.

Leibold, Mathew A. (1996). "A Graphical Model of Keystone Predators in Food Webs: Trophic Regulation of Abundance, Incidence, and Diversity Patterns in Communities". In: *The American Naturalist* 147.5, pp. 784–812. ISSN: 00030147, 15375323. URL: http://www.jstor.org/stable/2463090.

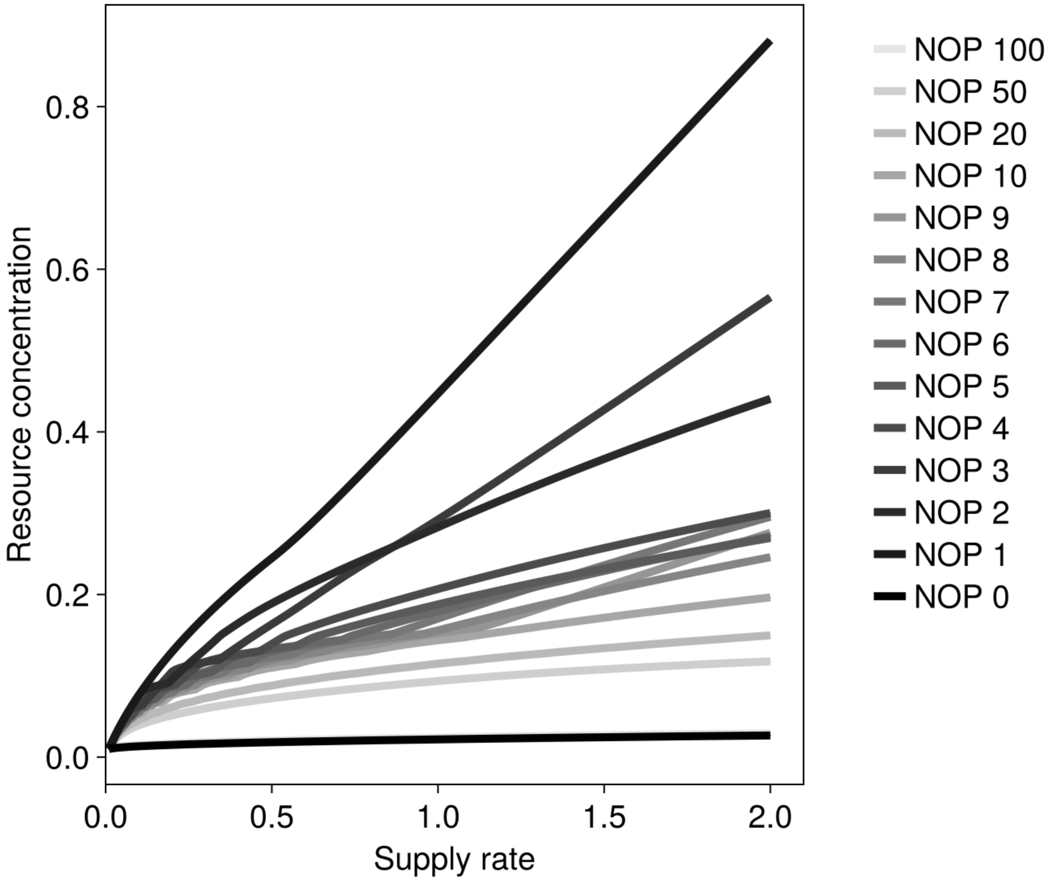

Fig.S 3: Resource concentration increases with the supply rate s under different food web structure with different number of predators.

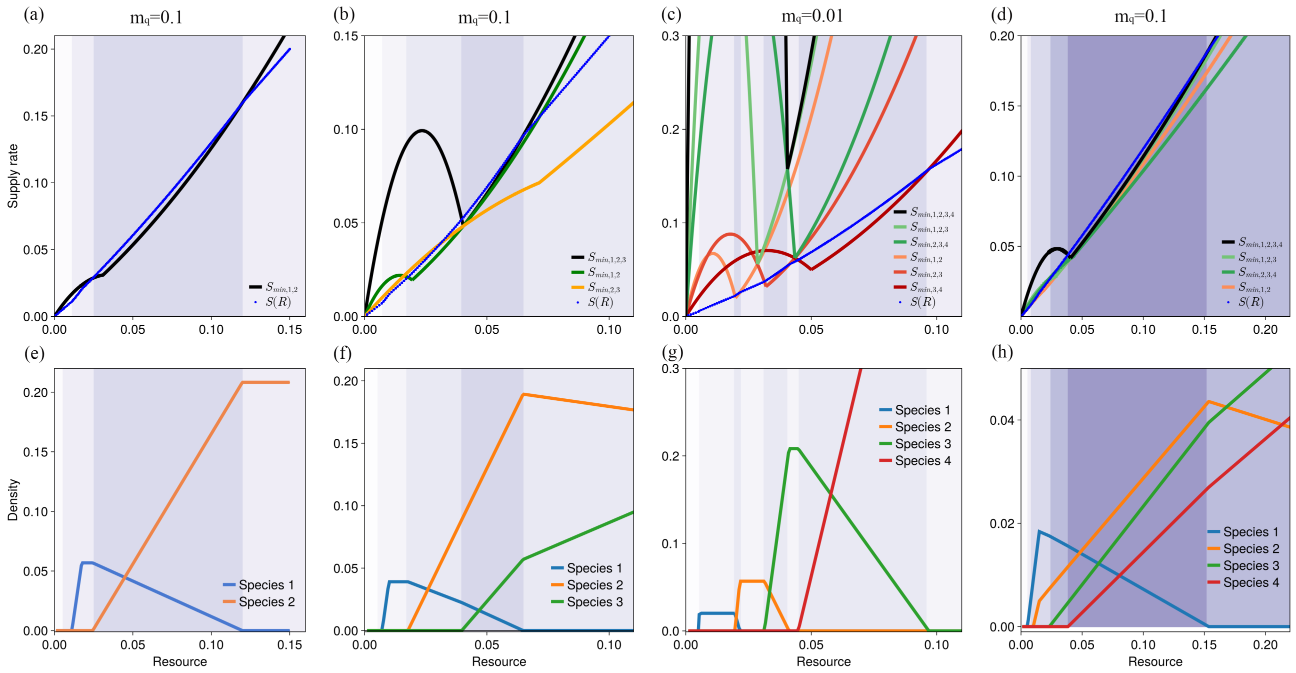

Fig.S 4: The criteria of the supply rate for species coexistence and the simulated density-resource curves.

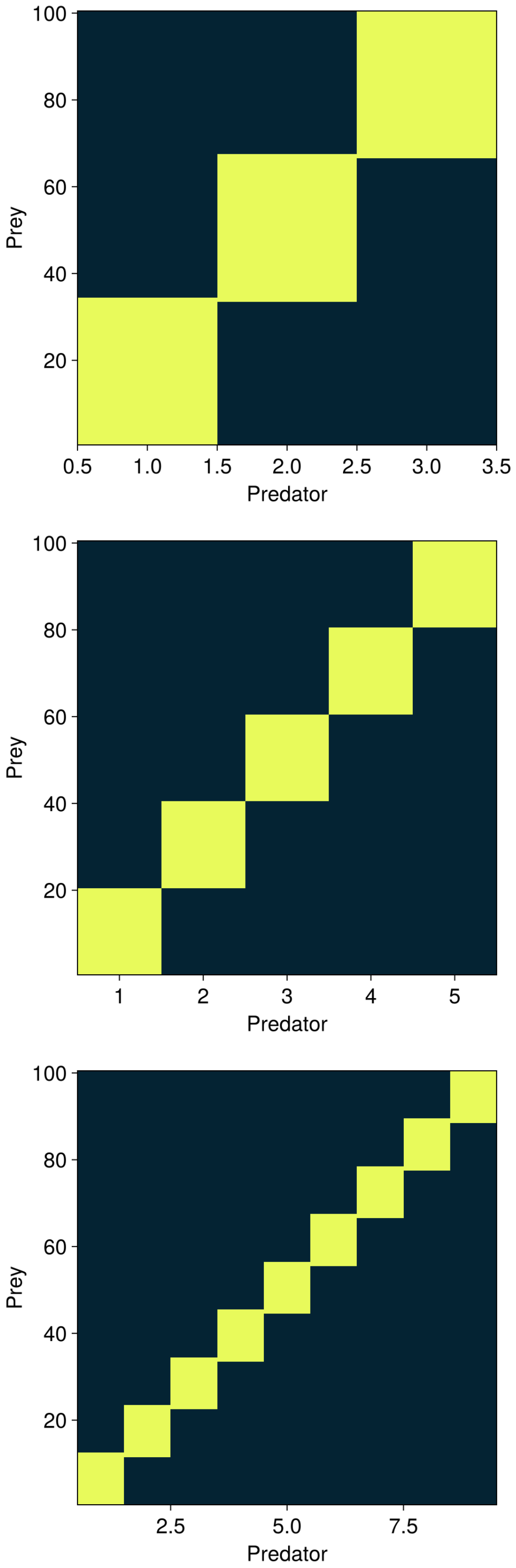

Fig.S 5: The predation matrix for three food webs with 3, 5, 9 predators. The predators target at different groups of prey with limited overlap. This assumption may reflect size-based predation with maximum growth rates being correlated to cell sizes in microbes.

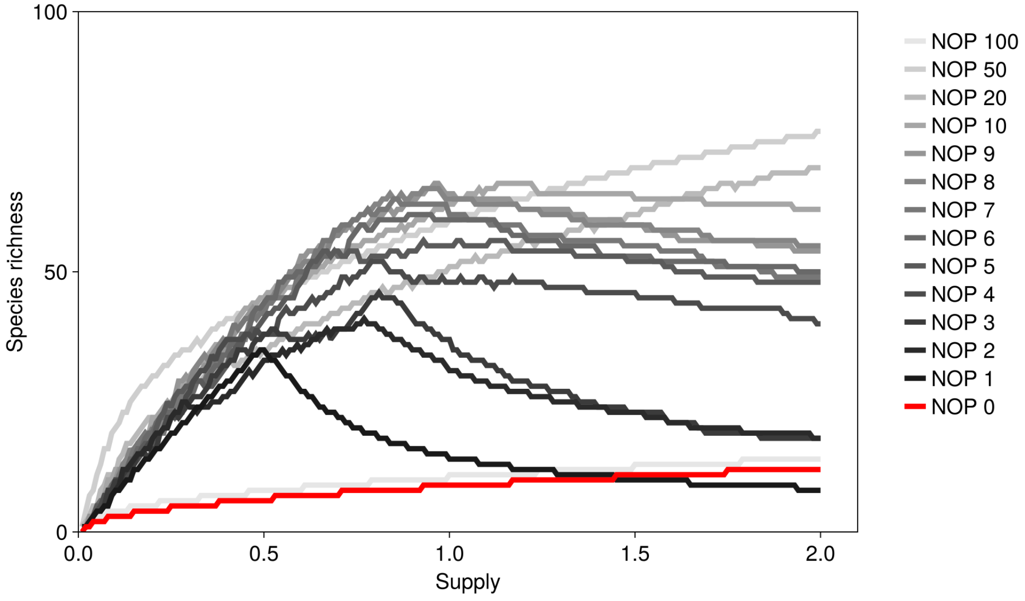

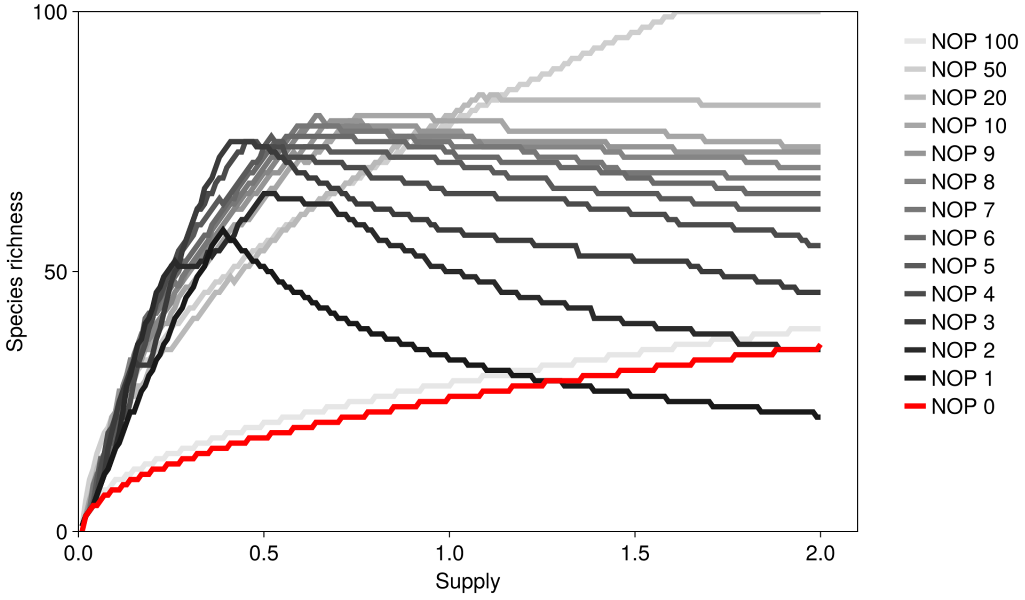

Fig.S 6: The PDRs under $m_q = 0.1, 10$.
